# Metabolomics reveals ascorbic acid inhibits ferroptosis in hepatocytes and boosts the effectiveness of anti-PD1 immunotherapy in hepatocellular carcinoma

**DOI:** 10.1186/s12935-024-03342-0

**Published:** 2024-05-31

**Authors:** Guoqiang Sun, Chuan Liu, Zhengqing Lu, Jinyu Zhang, Hengsong Cao, Tian Huang, Mingrui Dai, Hanyuan Liu, Tingting Feng, Weiwei Tang, Yongxiang Xia

**Affiliations:** 1 Hepatobiliary Center, The First Affiliated Hospital of Nanjing Medical University; Key Laboratory of Liver Transplantation, Chinese Academy of Medical Sciences, NHC Key laboratory of Hepatobiliary cancers, Nanjing, Jiangsu China; 2 Department of General Surgery, Nanjing First Hospital, Nanjing Medical University, Nanjing, Jiangsu China; 3 Central Laboratory, The Fourth Affiliated Hospital of Nanjing Medical University, Nanjing, Jiangsu China; 4 Stomatological college of Nanjing Medical University, Nanjing, China

**Keywords:** Ascorbic acid, Liver injury, Ferroptosis, Anti-PD1, Hepatocellular carcinoma, Metabolomics analysis

## Abstract

**Background:**

Immunotherapy combined with molecular targeted therapy is increasingly popular in patients with advanced hepatocellular carcinoma (HCC). However, immune-related adverse events(irAEs) brought on by immunotherapy increase the likelihood of side effects, thus it is important to look into ways to address this issue.

**Methods:**

Different metabolite patterns were established by analyzing metabolomics data in liver tissue samples from 10 patients(divided into severe and mild liver injury) before and after immuno-targeted therapy. After establishing a subcutaneous tumor model of HCC, the mice were divided into PBS group, ascorbic acid(AA) group, and anti-PD1 + tyrosine kinase inhibitor (TKI) group, anti-PD1 + TKI + AA group. Liver tissue were stained with hematoxylin-eosin staining(HE) and the content of aspartate transaminase (AST) and alanine transaminase(ALT) in blood were determined. The mechanism was confirmed by western blotting, mass cytometry, and other techniques.

**Results:**

Through metabolomics analysis, AA was significantly reduced in the sample of patients with severe liver injury caused by immuno-targeted therapy compared to patients with mild liver injury. The addition of AA in vivo experiments demonstrated a reduction in liver injury in mice. In the liver tissues of the anti-PD1 + TKI + AA group, the protein expressions of SLC7A11,GPX4 and the level of glutathione(GSH) were found to be higher compared to the anti-PD1 + TKI group. Mass cytometry analysis revealed a significant increase in the CD11b^+^CD44^+^ PD-L1^+^ cell population in the AA group when compared to the PBS group.

**Conclusions:**

AA could reduce liver injury by preventing hepatocyte SLC7A11/GPX4 ferroptosis and improve the immunotherapy effect of anti-PD1 by boosting CD11b^+^CD44^+^PD-L1^+^cell population in HCC.

**Supplementary Information:**

The online version contains supplementary material available at 10.1186/s12935-024-03342-0.

## Introduction

According to global cancer statistics, there are about 900,000 new liver cancer patients and 830,000 deaths in 2020, making it the sixth most common cancer and the third leading cause of cancer death worldwide. China has been the country with the heaviest burden of liver cancer in the world, accounting for 45.3% of new cases of liver cancer and 47.1% of deaths [1]. Unfortunately, the five-year survival rate of hepatocellular carcinoma (HCC), accounting for most of primary liver cancer patients, in China is only 12.1%. Although the five-year survival rate of HCC patients with surgical treatment can be improved to 60% [2], only 30% of patients with BCLC stage C who are initially diagnosed in China account for 55% can receive surgical treatment [3]. In comparison to other types of tumors, the five-year survival rate for HCC has not significantly improved in recent years, and the main challenges in HCC treatment continue to be postoperative metastasis and recurrence.

In recent years, there has been significant progress in the field of tumor immunotherapy, particularly in the use of combination immuno-targeted therapy as a common treatment for advanced HCC. The combination of PD1 monoclonal antibody (anti-PD1) and tyrosine kinase inhibitor (TKI) has shown promising initial results in liver cancer, and it has been included in the Chinese expert consensus on translational therapy of liver cancer (2021) [4]. However, while providing hope for patients, the administration of these preoperative drugs can lead to varying degrees of liver damage. Therefore, further research is needed to explore effective strategies for managing liver injury caused by immuno-targeted therapy.

Metabolomics, through modern analytical techniques, analyzes the changes of metabolites in different states of organisms, thus revealing the functional state of the organism as a whole. As a new omics, metabolomics is widely used in clinical and basic studies of liver injury. Metabolomics analysis of the effects of tretinoin on liver injury in mice has revealed that metabolites such as glycerophospholipids and fatty acids play important roles in the process of liver injury in mice [5]. Serum metabolite and cytokine levels in patients with drug-induced liver injury (DILI) have been assessed by metabolomics and cytokine analyses, and 31 metabolites and 5 cytokines are found to be strongly associated with the severity of DILI [6]. Hence, the analysis of metabolomics can aid researchers in comprehending the variations in metabolites among different causes of liver injury. It also demonstrates that metabolomics plays a crucial role in understanding the pathophysiology, pathway mechanism, occurrence, and progression of liver injury. There is increasing evidence that the activation of inhibitory immune checkpoint proteins such as PD1 can induce metabolic reprogramming of immune cells and cancer cells, thus influencing the nutritional competition between these two types of cells in the tumor microenvironment. PD1 activation can inhibit transcription coactivator PGC1α and control mitochondrial biogenesis [7]. In exhausted CD8^+^ T cells, PD1 receptor is involved in regulating early glycolysis and mitochondrial metabolic changes, leading to reduced glucose consumption and glycolysis rate, resulting in irreversible mitochondrial dysfunction [8]. In addition, PD1 can promote fatty acid β oxidation of endogenous lipids in activated T cells by increasing CPT1A expression [9]. In 116 stage IV melanoma patients treated with anti-PD1 antibodies, the functional differences between the responding and non-responding groups are mainly related to oxidation and lipid metabolism [10]. However, current studies mainly focus on the effects of PD1 monoclonal antibody on tumor cell metabolism, while there are few studies on normal liver damage. Therefore, it is an extremely innovative entry point to investigate the damage of normal liver caused by immuno-targeted therapy from metabolomics.

Based on the previous clinical trials published by our research group [11], we intended to find the causes of liver injury in HCC patients caused by immuno-targeted therapy from the perspective of metabolism. In the present study, we detected 20 liver samples of 10 HCC patients before and after immuno-targeted treatment by metabolomics analysis, and established metabolomics difference maps. We found that ascorbic acid(AA) significantly decreased in liver tissues of HCC patients after immuno-targeted treatment. Further analysis revealed that the decrease in AA was more pronounced in the group of patients with severe liver injury compared to those with mild liver injury. In addition, we confirmed that AA attenuates liver injury via SLC7A11/GPX4 ferroptosis pathway and enhances sensitivity to anti-PD1 therapy by boosting CD11b^+^CD44^+^PD-L1^+^ cell cluster in HCC in vitro and vivo.

## Materials and methods

### Cases and tissue specimen collection

Based on declaration of Helsinki, we made the informing process with respect to the present study. 20 liver samples from 10 HCC patients in First Affiliated Hospital of Nanjing Medical University, before and after immuno-targeted therapy was applied with metabolic sequencing. The collection of human specimens gained the approval from the Medical Ethics Committee of Nanjing Medical University. The overall samples received the efficiently collection after being removed from the body. According to the degree of liver injury, 10 patients were divided into severe liver injury group and mild liver injury group, with 5 patients in each group. After treatment, Aspartate transaminase (AST) and alanine transaminase(ALT) increased by more than 5 times than before treatment is for severe liver injury, and less than 5 times and higher than normal value is for mild liver injury. AT Group: severe liver injury group after target-immunotherapy, AC group: severe liver injury group before target-immunotherapy, BT group: mild liver injury group after target-immunotherapy, BC group: mild injury group before target-immunotherapy.

### Metabolomic analysis

Metabolites were isolated by ultrasonic shaking for one hour in ice baths with 1 mL precooled solutions of methanol, acetonitrile, and water. The mixture was then centrifuged at 14,000 g for 20 min. at 4 °C and left at -20 °C for 1 h. The supernatants were collected and concentrated in a vacuum until dry. A UPLC-ESI-Q-Orbitrap-MS system (UHPLC, Shimadzu Nexera X2 LC-30AD, Shimadzu, Japan) in conjunction with Q-Exactive Plus was used to evaluate the metabolomics profiling (Thermo Scientific, San Jose, USA). Samples were examined utilizing a 2.1 mm*100 mm ACQUIY UPLC BEH Amide 1.7 μm column(Waters, Ireland) for hydrophilic interaction liquid chromatography (HILIC) separation. The mobile phase contains A: 25 mM ammonium acetate and 25 mM ammonium hydroxide in water, and B: 100% acetonitrile. The flow rate was 0.5 mL/min (ACN). The gradient started off at 95% B and was linearly dropped to 65% in 7 min. It was then maintained for 1 min before being reduced to 35% in 2 min. It was then increased to 95% in 0.5 min with a 2 min re-equilibration interval. For MS data acquisition, both the positive-mode and the negative-mode of electrospray ionization (ESI) were used. Aliquots of all samples that were representative of the samples being analyzed were pooled to create quality control (QC) samples, which were then used for data normalization. QC samples and blank samples (75% ACN in water) were injected every six samples throughout the acquisition.

The variable influence on projection (VIP) values from the OPLS-DA model and a two-tailed Student’s t test (p value) on the normalized raw data at the univariate analysis level were used to determine the discriminating metabolites. One-way analysis of variance (ANOVA) for multiple groups analysis was used to determine the p value. Statistics-significant metabolites were those with VIP values greater than 1.0 and a p value lower than 0.05. The average mass response ratio between two arbitrary classes was used to calculate fold change. On the other hand, cluster analyses using the R package were carried out using the identified differential metabolites. The differential metabolite data were subjected to KEGG pathway analysis using the KEGG database to identify the perturbed biological pathways. The Fisher’s exact test and FDR correction for multiple testing were used in KEGG enrichment analyses.

### Cell culture

Hepa1-6 and Lo2 cells were made available by the Cell Bank of Type Culture Collection (Chinese Academy of Sciences, China), culturing with RPMI 1640 medium (BI, USA) supplemented and 10% fetal bovine serum (FBS) (Gibco, USA) at 37 °C in a 5% CO_2_ chamber. In a single incubator with a constant temperature and 5% CO_2_, maintenance was done on all cell lines. The temperature was 37 °C.

### Fluorescence probe assay of reactive oxygen species(ROS)

Lo2 cells were cultured overnight at 37 °C in a 5% CO2 incubator after being seeded in an 8-well plate (Corning, USA). The 10mmol/l carbon tetrachloride (CCL_4_) group, the 10mmol/l CCL4 + 50µmol/l AA group, and the control group were also cultured overnight at 37 °C in a 5% CO_2_ incubator. The medium was then removed, and the cells were washed twice with 200 ul Hank’s Balanced Salt Solution (HBSS). After removing HBSS, 200 ul of 1 µmol/l Liperfluo (Dojindo, Japan, Cat#L248) diluted with HBSS was added. The solution was incubated at 37 °C in a 5% CO_2_ incubator for 30 min. After incubation, the solution was removed, and the cells were washed twice with 200 µl HBSS. Finally, the cells were observed using a laser confocal fluorescence microscope.

### AST and ALT measurement

In the presence of excess ethylenediaminetetraacetic acid (EDTA), blood was drawn from the heart or tail vein, centrifuged, and analyzed using a commercial kit in accordance with the manufacturer’s protocol (Rsbio, China).

### Glutathione(GSH) measurement

A commercial kit (Nanjing Jiancheng Bioengineering Institute, China, Cat#A005-1-2) was used to measure GSH. In a solution of 5% metaphosphoric acid/0.6% sulfosalicylic acid, tissues were weighed and homogenized. The amount of total glutathione was calculated as µmmol/g tissue using a glutathione reductase and Nicotinamide Adenine Dinucleotide Phosphate(NADPH)-coupled reaction with 5, 50-dithiobis.

### AA measurement

The tissue was weighed, homogenized according to the ratio of weight (g): volume (ml) = 1:9, and centrifuged to obtain supernatant.0.1 ml tissue homogenate was taken, mixed with reagents, centrifuged to obtain supernatant, which was made into supernatant. According to the instructions (Nanjing Institute of Jiancheng Bioengineering, China, Cat#A009-1-1), the colorimetric determination was finally carried out at 536 nm wavelength and 1 cm optical path.

### Western blotting

Proteins were extracted from the cell and tissue using RIPA buffer (Sigma-Aldrich, USA, Cat#R0278), resolved by SDS-polyacrylamide gel, and then transferred to PVDF membranes. The primary antibodies (Abcam, UK) against SLC7A11,GPX4, β-ACTIN were used. Peroxidase-conjugated secondary antibody (CST, Sigma-Aldrich, USA) was used, and the antigen-antibody reaction was visualized by enhanced chemiluminescence assay (ECL; Thermo Fisher, USA).

### Hematoxylin-eosin (HE) and immunohistochemistry(IHC)

The sections were stained for several minutes in hematoxylin aqueous solution to detect HE. The color separation was briefly exposed to ammonia water and acid water, respectively. Rinse for one hour with water, and then add some distilled water and let it sit. Dehydrate in 70% alcohol for a further 10 min each. 2 min of alcohol eosin staining solution. Pure alcohol was used to dehydrate the stained section, and xylene was used to make it transparent. Gum was dropped on the transparent section, covering the cover glass seal. Slice specimens could be used after a label was applied and the gum had slightly dried. For immunohistochemistry, sections that had paraffin embedded in them underwent the deparaffinizing and rehydrating procedures. To stop peroxidase activity, hydrogen peroxide was used. At 4 °C, sections were incubated all night long with the primary antibody (PD1, Ki67, PD-L1, CD11b, CD44; Abcam, UK). After treating tissue sections with the biotinylated secondary antibody, streptavidin-horseradish peroxidase complex was added (Santa Cruz Biotechnology Inc., USA). The Knodell score was used to determine the severity of the necroinflammatory process. The interface inflammation scoring system was scored on a scale of 0–10 for the degree of hepatocellular degeneration and necrosis; the inflammatory state of the intralobular confluent area was scored on a scale of 0–4, and the degree of fibrosis was scored on a scale of 1–4.

### Immunofluorescence(IF)

Samples that had been embedded in paraffin were sectioned at a 4 mm thickness. Antigen retrieval was achieved by pressure boiling for 3 min in 0.01 mol/L citrate buffer. After being fixed with 4% paraformaldehyde for 20 min at room temperature, immunofluorescence tissue was permeabilized with 0.05% Triton X-100 (Sigma-Aldrich, USA) in PBS for 5 min. Antibodies against CD11b (1:200; Abcam, UK), CD44 (1:200; Abcam, UK), PD-L1 (1:200; Abcam, UK)were incubated with the samples for a whole night at 4 °C. After that, samples were incubated for 1 h at room temperature with Alexa Fluor or HRP-conjugated secondary antibodies. DAPI (Sigma-Aldrich, USA) was used to counterstain the nuclei, and laser scanning confocal microscopy was employed to take pictures (Zeiss, Germany).

### Subcutaneous tumor model of HCC

The animal experiment conducted by Nanjing Medical University’s animal management committee was approved, and all experimental procedures and animal care followed the ethical guidelines set by the institution for animal-related experiments. The experiment was granted ethical approval with the number 2017-SRFA-138. The mice used in the experiment were purchased from Charles River. To euthanize the mice, cervical dislocation was performed. Prior to the procedure, the mice were anesthetized using isoflurane. The mice were placed on a wire mesh, and the experimenters firmly held the tail with one hand while applying pressure on the head with the thumb and index finger of the other hand to quickly dislocate the cervical vertebrae. This method ensured rapid loss of consciousness and death in the mice.

The injection of Hepa1-6 cells was subcutaneously made into 6–8 week C57BL/6 female mice. Carcinoma transplanted tumor model mice fell to four groups, PBS, AA (MCE, USA), anti-PD1 (bioxcell, USA) + TKI(MCE, USA), anti-PD1 + TKI + AA, with 5 mice in the respective group. On the ninth day and every two days after that, 150ul of 1.5 M AA was injected intraperitoneally, we adjusted to this dose as appropriate [12]; on the eighth day and every four days after that until sixteenth day, 6.6 mg/kg of anti-PD1 was injected intraperitoneally [13]; Fer-1(Sigma, USA) at a dose of 5 mg/kg, and anti-PD1 was injected once intraperitoneally 1 h before injection [14]; on the sixth day and every four days after that until fourteenth day; 150 mg/kg Apatinib (Jiangsu Hengrui Medicine Co., Ltd. China) were administered via oral gavage [15]. The activity, spirit, and diet of the mice in each of the aforementioned mouse models were tracked daily before and after the experiment. Every four days, vernier calipers were used to measure the tumor’s long diameter (mm) and short diameter (mm). The tumor volume (V) of the mice was then calculated using the formula V = ab^2^/2, and the tumor growth curve was plotted. The mice were euthanized on day 20, and tumor samples were examined for further investigation.

### Mass cytometry

The tissue samples were obtained from two distinct groups of C57BL/6 mice: PBS and AA. The CyTOF staining process consisted of several steps, namely 194Pt staining, Fc block staining, surface antibody staining, overnight DNA staining using 191/193Ir, intracellular antibody staining, and data collection on a computer. The data analysis procedure involved the following steps: A. FlowJo pretreatment: Identifying and selecting individual, fully functional CD45^+^ immune cells by encircling them. Bio-information analysis: Using the X-shift algorithm for cell subpopulation clustering, manual annotation, TSNE dimensionality reduction visual display, and statistical analysis.

### Statistical analysis

GraphPad Prism 8.0 (GraphPad, USA) was used for our statistical analyses, and a P value of 0.05 or lower was regarded as statistically significant. For the purpose of comparing continuous variables between the two groups, an independent t-test was conducted.

## Results

### Map of metabolic differences in liver tissue samples of HCC patients before and after immuno-targeted therapy

**Fig. 1:**
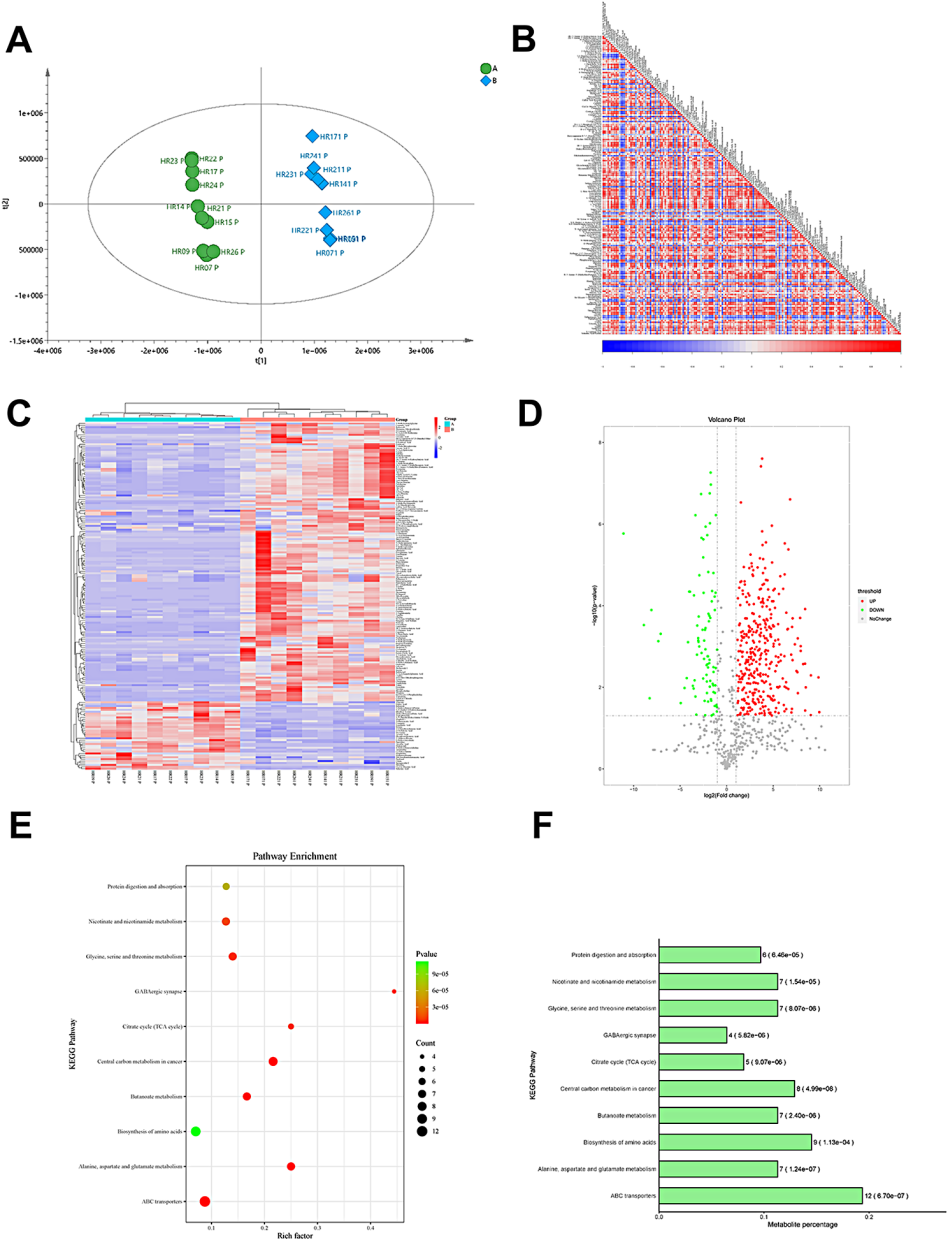
Map of metabolic differences in liver tissue samples of HCC patients before and after immuno-targeted therapy. (**A**) PLS-DA method was used to observe the overall distribution trend of all samples. R2X and R2Y denote the percentage of X and Y matrix information that can be explained by the PLS-DA classification model, respectively, and Q2Y is calculated by cross-validation. R2X = 0.473, R2Y = 0.998,Q2 = 0.983 (**B**) Based on the Pearson correlation analysis method, the correlation coefficient (R) between the differential metabolites was between − 1 and + 1. *R* > 0 means positive correlation, which is represented by red; *R* < 0 means negative correlation, which is represented by blue. (**C**) The results of hierarchical clustering of differential metabolites caused by liver injury samples before(A group) and after(B group) treatment. The redder the color, the higher the relative expression level, the bluer the lower the relative expression level. (**D**) Volcano plot, with FC > 1.5 or FC < 0.667, and P value < 0.05 as the screening criteria, the vertical dotted line analysis indicates log2(1/1.5) and log2(1.5), red dots in the figure indicate up-regulated metabolites, blue Dots indicate down-regulated metabolites.(**E**) The significant pathway bubble map shows the top10 significant pathways. (**F**) Histogram of KEGG pathway enriched for significant top10

We detected 20 liver samples of 10 HCC patients before and after immuno-targeted treatment by metabolomics analysis, and established metabolomics difference maps. We performed partial least squares discrimination analysis(PLS-DA) of metabolites before and after treatment to reflect the variation between and within the sample groups in general. PLS-DA method was used to observe the overall distribution trend of all samples, and the results showed separation of pretherapy and post-treatment (Fig. 1A). The explanatory rate R2Y = 0.998 and predictive rate Q2 = 0.983 of the PLS-DA analysis, both of which were close to 1, indicated that this PLS-DA model explained and predicted the differences between the two groups of samples well, representing a good predictive ability of the model. Based on Pearson correlation analysis method, the correlation coefficients between significantly different metabolites were calculated and presented in the form of correlation coefficient matrix heat map(Fig. 1B). Heatmap plot was used to show differential metabolites before and after immuno-targeted treatment and results showed that most metabolites were altered in before immuno-targeted treatment relative to after immuno-targeted treatment. The elevated ranked top 20 metabolites included Perfluoropropanesulfonic Acid, Secopenitrem D, N-Acetylneuraminate, Fenoldopam, 2,4-Dinitrotoluene, Etomidate, Gly-Val, Pangamic Acid Sodium, 8-Gingerol, D-(+)-Malic Acid, Destruxin A, Serine-Cholic Acid, Toradol, D-(+)-Pantothenic Acid, Butyrolactone I, 2-Coumaric Acid, Norharman, Raltitrexed, Xanthyletin, N-Acetyl-Dl-Methionine. Meanwhile, there were top 20 decreased metabolites, contained Isopalmitic Acid, Dodemorph, Delsoline, 3-Hydroxybenzo(A)Pyrene, N,N-Dimethyldodecylamine N-Oxide, Ascorbic Acid, Menthol, Styrene, Morpholine, Pacifenol, 6-Hydroxynicotinate, 3,4-Dimethoxybenzoic Acid, Cis, Cis-Muconic Acid, Maleic Acid, Methanesulfonate, Triptophenolide, Dodecylbenzenesulfonic Acid, Rhodinyl Acetate, 4-Hydroxybenzoylcholine, Cyclohexylamine (Fig. 1C and D). KEGG pathway analysis showed that these differential metabolites were mainly concentrated in central carbon metabolism in cancer, alanine, aspartate and glutamate metabolism, ABC transporters, butanoate metabolism, glycine, serine and threonine metabolism, citrate cycle (TCA cycle), nicotinate and nicotinamide metabolism, protein digestion and absorption, and biosynthesis of amino acids (Fig. 1E and F).

### Map of metabolic differences in liver tissue samples with severe liver injury before and after immuno-targeted therapy

**Fig. 2:**
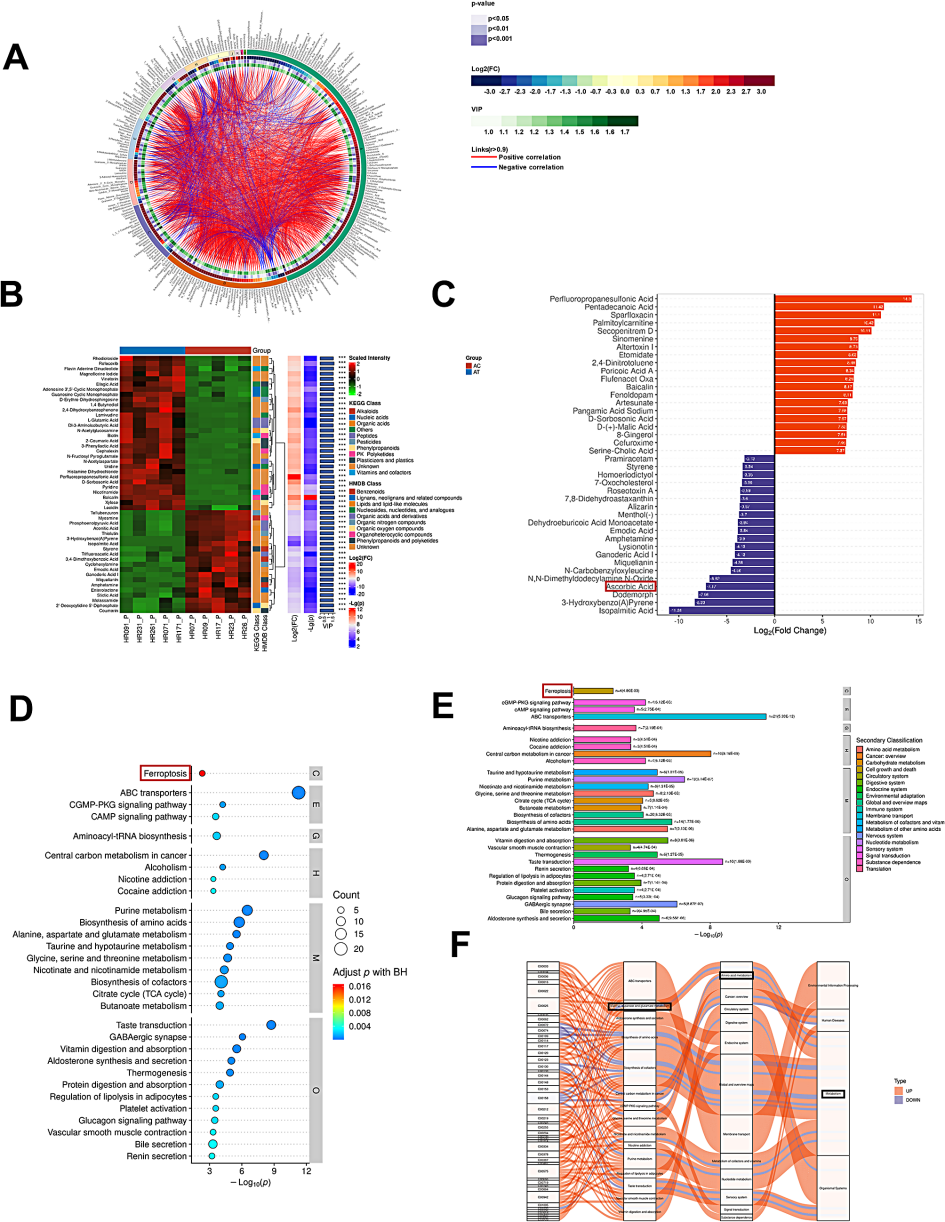
Map of metabolic differences in liver tissue samples with severe liver injury before and after immuno-targeted therapy. (**A**) The circos diagram of differential metabolite. (**B**) Differential metabolite hierarchical clustering results, the redder the color, the higher the relative expression, and the bluer the bluer, the lower the relative expression. The metabolite classification information of the KEGG and HMDB databases is also shown in the figure. (**C**) Differential metabolites with a higher degree of importance are displayed, the abscissa is the log transformation of FC, and the ordinate is the metabolites. The blue and red dots on the left and right sides represent the down-regulated and up-regulated differential metabolites, respectively. (**D**) The pathways with the top30 significance are displayed, the abscissa represents the negative logarithmic transformation of p-value, the ordinate represents the name of the pathway, and the size of the circle represents count, that is, the number of differential metabolites annotated into the pathway; the color of the circle corresponds to the corrected p -value, more significant from red to blue. (**E**) Salient pathway histogram showing KEGG pathway enriched for salience top30. (**F**) The first column on the left of the significant pathway Sankey diagram represents the differential metabolites of up & down, and the width of the branch curve corresponds to the size of the data flow

We analyzed the changes of metabolites in 5 HCC patients with severe liver injury before and after immuno-targeted treatment. We adopted positive, negative, and mix mode to analyze the differential metabolites respectively. The mix model was applied firstly. The circos diagram mainly showed the correlation between multiple differential metabolites. The following combinations were among the top 10 differential metabolites that we examined at in our analysis, including Baicalin, Pyridine, Nicotinamide, Adenosine.3`,5`-Cyclic Monophosphate, Perfluoropropanesulfonic Acid, Rofecoxib, Phosphoenolpyruvic Acid, Aconitic Acid, Styrene, and D-Sorbosonic Acid (Fig. 2A). According to the structure and function of metabolites, the different metabolites in each comparison group were classified and counted, and the results of substance classification in KEGG and HMDB databases were provided respectively(Fig. 2B). The top 20 up-regulated metabolites(including Perfluoropropanesulfonic Acid, Pentadecanoic Acid, Sparfloxacin, Palmitoylcarnitine, et al.) and the top 20 down-regulated metabolites (including Pramiracetam, Styrene, Homoeriodictyol, 7-Oxocholesterol, Ascorbic Acid, et al.) were shown Fig. 2C according to the multiples of difference. KEGG pathway analysis showed that these differential metabolites were mainly concentrated in ferroptosis, ABC transporters, biosynthesis of cofactors, biosynthesis of amino acids, purine metabolism, vitamin digestion and absorption, et al. (Figure 2D and E). Sankey diagram was used to visually analyze the trend of data flow between downregulated metabolites and various pathways and results indicated that the rightmost hierarchy with the largest pool of differential metabolites is metabolism, and the most concentrated in the metabolic pathway is global and overview maps of biosynthesis of amino acids and cofactors. And glutamate metabolism accounted for half of the amino acids metabolism(Fig. 2F). Next, both positive and negative patterns were analyzed, and the results were shown in Figure S1-S2. The above results showed that the metabolism of liver tissue samples with severe liver injury was significantly different before and after immuno-targeted therapy.

### Map of metabolic differences in liver tissue samples with mild liver injury before and after immuno-targeted therapy

**Fig. 3:**
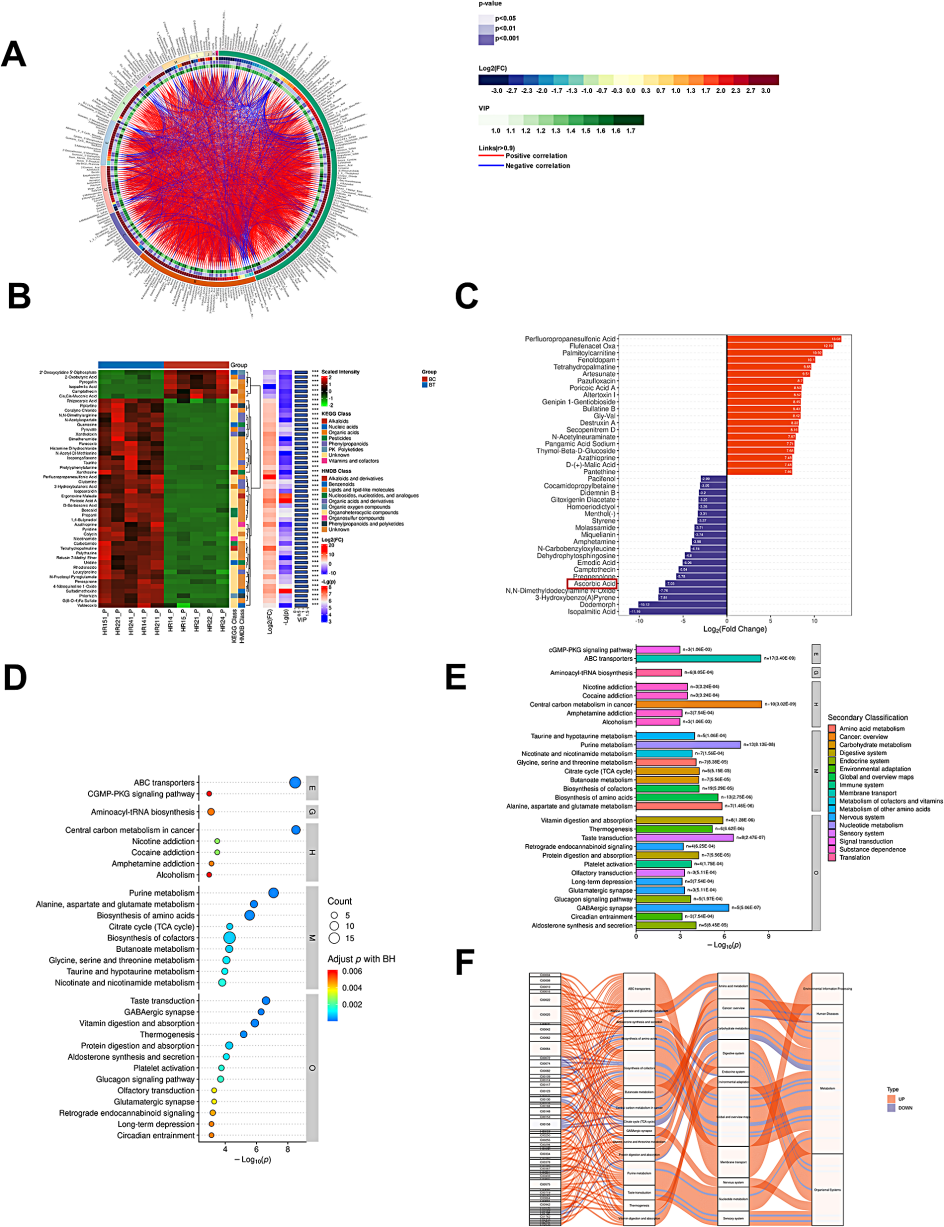
Map of metabolic differences in liver tissue samples with mild liver injury before and after immuno-targeted therapy.(**A**) The circos diagram of differential metabolite. (**B**) Differential metabolite hierarchical clustering analysis (**C**) Differential Metabolite Importance Analysis (**D**) The prominent top30 pathway salient pathway bubble chart (**E**) Salient pathway histogram showing KEGG pathway enriched for salience top30. (**F**) Significant pathway Sankey diagram

We analyzed the changes of metabolites in 5 HCC patients with mild liver injury before and after immuno-targeted treatment. We adopted positive, negative, and mix mode to analyze the differential metabolites respectively. Firstly, the mix model was applied. We investigated the connection between the top 10 most important differential metabolites, which included Sulfadimethoxine, 4-Nitroquinoline 1-Oxide, Ergonovine Maleate, Sulfadimethoxine, Propanil, Boscalid, Pyruvate, Poricoic Acid A, Histamine Dihydrochloride and Guanosine (Fig. 3A). According to the structure and function of metabolites, the different metabolites in each comparison group were classified and counted, and the results of substance classification in KEGG and HMDB databases were provided respectively(Fig. 3B). The top 20 up-regulated metabolites(including Perfluoropropanesulfonic Acid, Flufenacet Oxa, Palmitoylcarnitine, et al.) and the top 20 down-regulated metabolites (including Pacifenol, Cocamidopropylbetaine, Didemnin B, Gitoxigenin Diacetate, Ascorbic Acid, et al.) were shown Fig. 3C according to the multiples of difference. KEGG pathway analysis showed that these differential metabolites were mainly concentrated in biosynthesis of cofactors, purine metabolism, biosynthesis of amino acids, central carbon metabolism in cancer, taste transduction, vitamin digestion and absorption, et al. (Figure 3D and E). Sankey diagram indicated that the rightmost hierarchy with the largest pool of differential metabolites is metabolism, and the most concentrated in the metabolic pathway is global and overview maps of biosynthesis of amino acids and cofactors. And glutamate metabolism accounted for half of the amino acids metabolism (Fig. 3F). Next, both positive and negative patterns were analyzed, and the results were shown in Figure S3-S4. These results indicate that even in mild liver injury, there are still many changes in the metabolism of liver tissue samples before and after immunotargeted therapy.

### AA attenuates liver injury in vivo and vitro

**Fig. 4:**
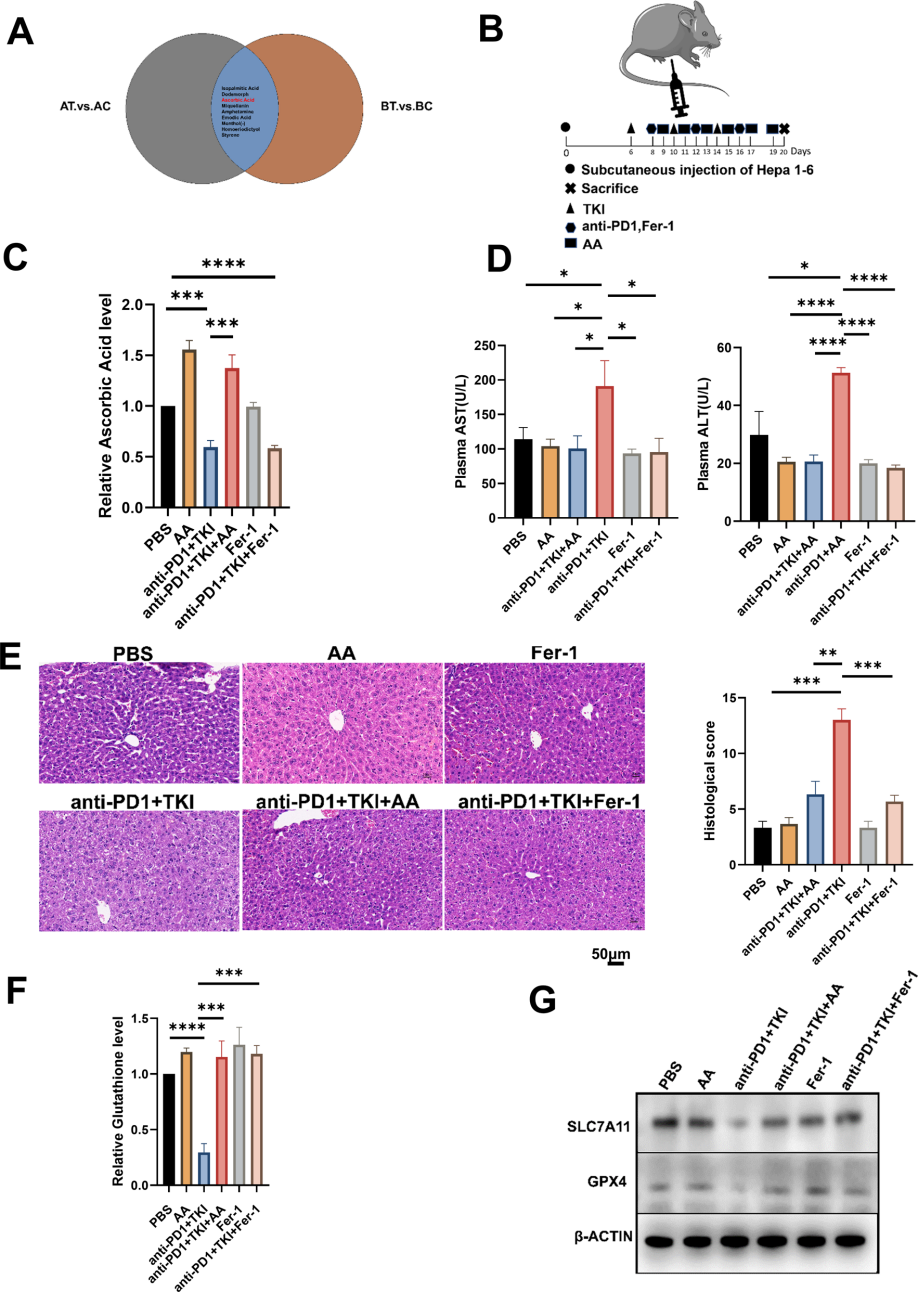
AA attenuates liver injury by modulating ferroptosis pathway in vivo. (**A**) Before and after immuno-targeted treatment, the same differential metabolites were found in the two groups of samples with mild(BT. vs. BC) and severe liver injury(AT. vs. AC). (**B**) Diagram of experimental steps in mice. (**C**) The content of AA in liver samples of mice in PBS group, AA group, anti-PD1 + TKI group, anti-PD1 + TKI + AA group, Fer-1 group, and anti-PD1 + TKI + Fer-1 group. (**D**) The content of ALT, AST in blood of mice in PBS group, AA group, anti-PD1 + TKI group, anti-PD1 + TKI + AA group, Fer-1 group, and anti-PD1 + TKI + Fer-1 group. (**E**) HE staining of mouse liver tissue and pathology score in PBS group, AA group, anti-PD1 + TKI group, anti-PD1 + TKI + AA group, Fer-1 group, and anti-PD1 + TKI + Fer-1 group. Scale bar, 50 μm.(**F**) The content of GSH in liver samples of mice in PBS group, AA group, anti-PD1 + TKI group, anti-PD1 + TKI + AA group, Fer-1 group, and anti-PD1 + TKI + Fer-1 group. (**G**) SLC7A11, GPX4, β-ACTIN protein expression in PBS group, AA group, anti-PD1 + TKI group, anti-PD1 + TKI + AA group, and Fer-1 group, and anti-PD1 + TKI + Fer-1 group. *, *P* < 0.05; **, *P* < 0.01; ***, *P* < 0.001,****, *P* < 0.0001

Overall, there were some consistent findings in the analysis of individuals with severe and mild liver damage. Perfluoropropanesulfonic Acid, Secopenitrem D, Fenoldopam, Pangamic Acid Sodium, D-(+)-Malic Acid, Menthol(-), and Styrene were all among the top 20 increasing metabolites identified in the three analysis. Isopalmitic Acid, Dodemorph, 3-Hydroxybenzo(A)Pyrene, N, N-Dimethyldodecylamine N-Oxide, AA(Ascorbic Acid) were shared among the top 20 descending metabolites in the three analyses(Fig. 4A). The KEGG pathway analysis of the overall analysis enriched in glutamate metabolism, biosynthesis of amino acids, the analysis of individuals with severe liver injury enriched in ferroptosis, biosynthesis of amino acids, vitamin digestion and absorption, and the analysis of individuals with mild liver injury enriched in biosynthesis of amino acids, vitamin digestion and absorption. Amino acid biosynthesis, glutamate metabolism, and vitamin digestion and absorption are all associated with ferroptosis [16, 17]. Moreover, the levels of AA were significantly decreased in the liver tissue that underwent treatment. Consequently, we would like to investigate the link between AA and ferroptosis in liver tissue.

**Fig. 5:**
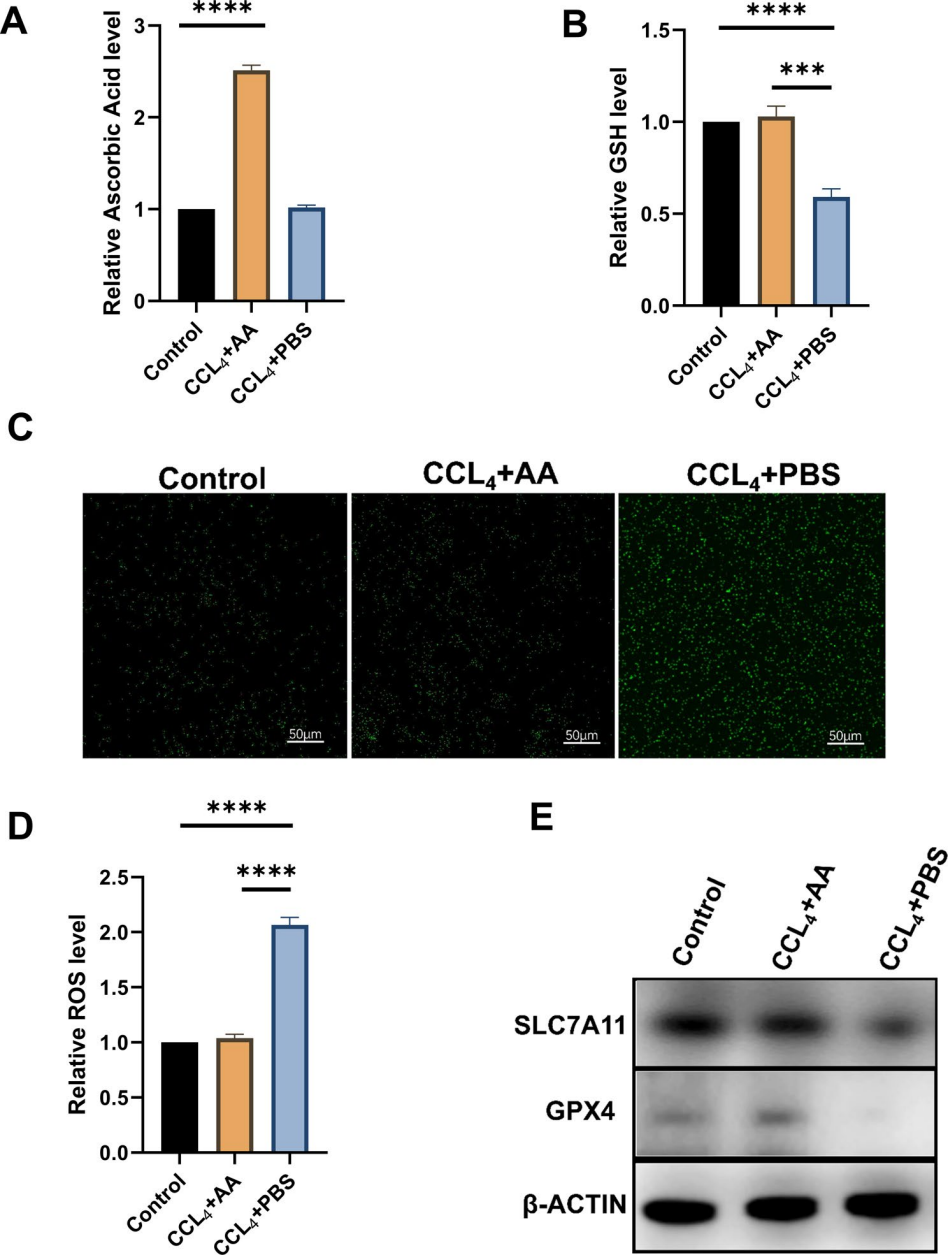
AA attenuates liver injury by modulating ferroptosis pathway in vitro. (**A**) The content of cellular AA in the control, CCL4 + PBS and CCL4 + AA groups. (**B**) The content of cellular GSH in the control, CCL4 + PBS and CCL4 + AA groups. (**C**) Fluorescent probes detect reactive oxygen specie(ROS) in control group, CCL_4_ + PBS group and CCL_4_ + AA group. (**D**) Relative ROS level in control group, CCL_4_ + PBS group and CCL_4_ + AA group. (**E**) SLC7A11, GPX4, β-ACTIN protein expression in control group, CCL_4_ + PBS group and CCL_4_ + AA group. **, *P* < 0.01; ***, *P* < 0.001,****, *P* < 0.0001

Through cross-analysis, we found that AA decreased significantly in the total differential metabolites before and after immuno-targeted treatment, and decreased more significantly in HCC patients with severe liver injury than in the group with mild liver injury. For addressing the possible effect exerted by AA for immuno-targeted-induced liver injury in HCC patients, we injected Hepa1-6 cells in C57BL/6 mice and treated the mice with PBS, AA, anti-PD1 + TKI, anti-PD1 + TKI + AA, respectively (Fig. 4B).We first detected the amount of AA and found that the content of AA in the anti-PD1 + TKI group was significantly lower than that in the PBS group, and the addition of AA increased the content(Fig. 4C).The results of liver enzyme showed that AST and ALT in anti-PD1 + TKI + AA group was significantly lower than that in anti-PD1 + TKI group (Fig. 4D). HE results of the liver showed that the liver injury of anti-PD1 + TKI + AA group was significantly lighter than that of anti-PD1 + TKI group, and pathology was evaluated using the Knodell score. (Fig. 4E). Fer-1 is a ferroptosis inhibitor that acts by inhibiting lipid peroxidation. We also established the Fer-1 group and the anti-PD1 + TKI + Fer-1 group to investigate whether the liver tissue injury induced by immuno-targeted treatment was caused by ferroptosis. Fer-1 reduced AST, ALT levels and attenuated liver injury caused by immuno-targeted treatment(Fig. 4C and E). Both AA and Fer-1 had the same effect of attenuating treatment-induced liver injury. In addition, we simulated liver injury by adding CCL_4_ to normal liver cells Lo2. The results showed the addition of AA increased the AA content in liver cells with CCL_4_ (Fig. 5A). These animal and cellular results suggested that AA could reduce immuno-targeted liver injury.

### AA attenuates immuno-targeted-induced liver injury by modulating SLC7A11/GPX4 ferroptosis pathway in HCC

However, the cause of AA attenuating immuno-targeted-induced liver injury is still unknown. We found that previous enrichment analysis were involved in ferroptosis, so we hypothesized that AA slowed the liver by inhibiting ferroptosis pathway. To prove our hypothesis, we examined the GSH content in liver of different groups of mice and found that the content of GSH in anti-PD1 + TKI group was significantly lower than that of PBS group, which was improved by the increase of AA and Fer-1(Fig. 4F). Western blotting confirmed that ferroptosis-related proteins including GPX4 and SLC7A11 proteins were both down-regulated in anti-PD1 + TKI group, indicating ferroptosis increased. Meanwhile, AA and Fer-1 could enhance expression of GPX4 and SCL7A11 proteins(Fig. 4G). In addition, we simulated liver injury by adding CCL_4_ to normal liver cells Lo2. The results showed the GSH content in liver cells with CCL_4_ + AA was significantly higher than that in CCL_4_ + PBS group (Fig. 5B). The ROS probe suggested that the ROS content in CCL_4_ + PBS group was significantly higher than that of the CCL_4_ + AA group (Fig. 5C and D). Western blotting confirmed that ferroptosis-related proteins were both up-regulated in CCL_4_ + AA group (Fig. 5E). These results demonstrated that AA alleviated immune-targeting induced liver injury by regulating the SLC7A11/GPX4 ferroptosis pathway in HCC.

### AA enhances anti-PD1 HCC therapeutic effect by increasing CD11b^+^ CD44^+^ PD-L1^+^cell population infiltration

**Fig. 6:**
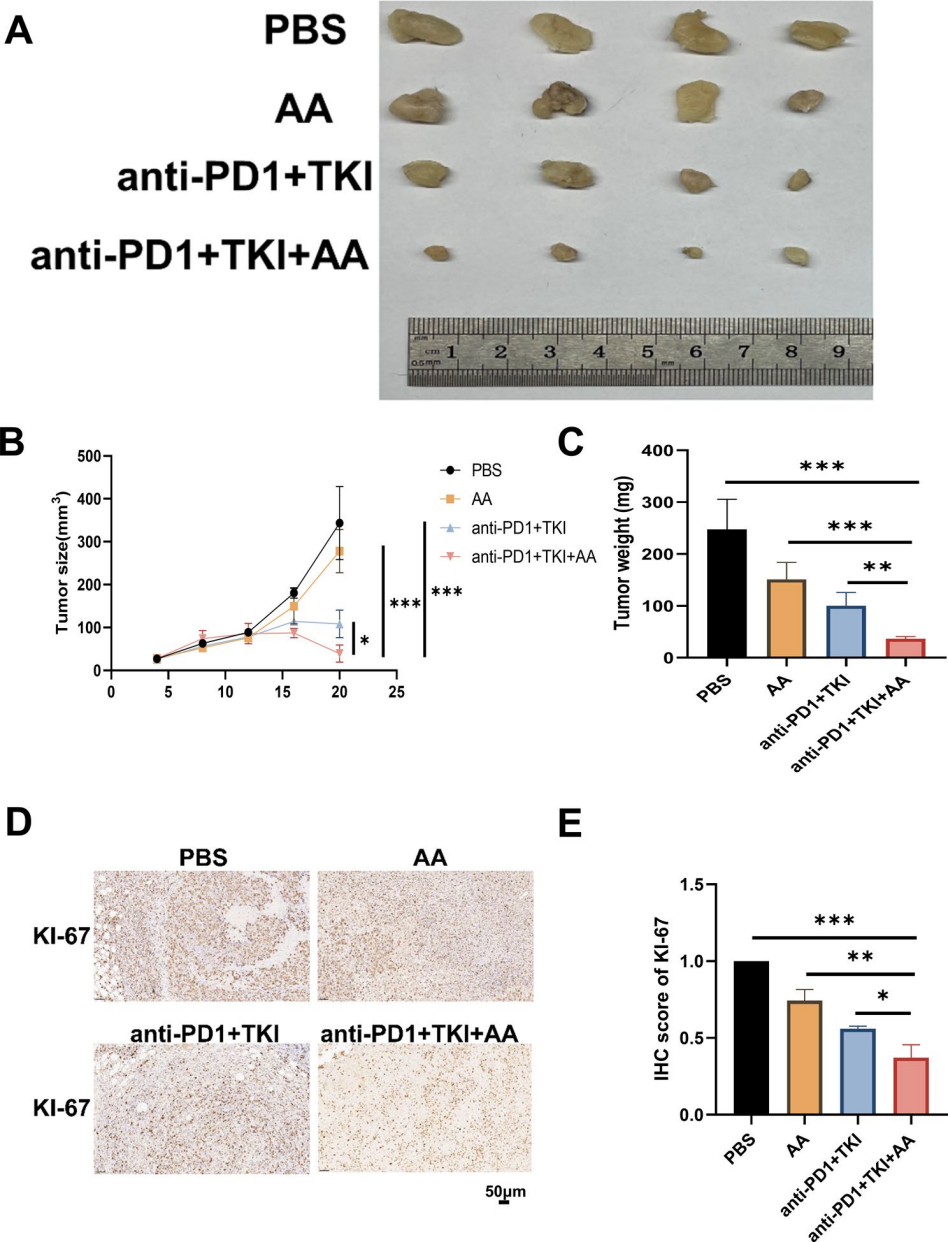
AA improves the efficacy of anti-PD1 therapy in HCC patients. (**A**) Mouse tumor pictures of PBS group, AA group, anti-PD1 + TKI group, and anti-PD1 + TKI + AA group. (**B**) Mouse tumor growth volume of PBS group, AA group, anti-PD1 + TKI group, and anti-PD1 + TKI + AA group. (**C**) Mouse tumor weight of PBS group, AA group, anti-PD1 + TKI group, and anti-PD1 + TKI + AA group. (**D**) Mouse tumor immunohistochemical pictures of PBS group, AA group, anti-PD1 + TKI group, and anti-PD1 + TKI + AA group. Scale bar, 50 μm. (**E**) Histogram of mouse tumor immunohistochemical analysis in PBS group, AA group, anti-PD1 + TKI group, and anti-PD1 + TKI + AA group. *, *P* < 0.05; **, *P* < 0.01; ***, *P* < 0.001

Since AA could alleviate liver injury, we needed to consider its effect on tumor growth to avoid its counterproductive effect. For addressing the possible effect exerted by AA for HCC tumors, we injected Hepa 1–6 cells in C57BL/6 mice and treated the mice with PBS, AA, anti-PD1 + TKI, anti-PD1 + TKI + AA, respectively. Hepa1-6 cells with PBS tended to grow faster in mice, whereas they showed the attenuation in AA or anti-PD1 + TKI group (Fig. 6A). When anti-PD1 was added on the 8th day in AA + anti-PD1 + TKI group, the tumors tended to grow more slowly (Fig. 6A).The mice were euthanized on day 20, and the volume and weight of the tumor in AA or anti-PD1 + TKI group were found significantly smaller than those in PBS group, and those in AA + anti-PD1 + TKI were much smaller than those in AA or anti-PD1 + TKI group (Fig. 6B). According to IHC results, compared with the PBS group, the expression of Ki67 was significantly decreased after AA was injected (Fig. 6D and E). In the anti-PD1 + TKI + AA group, Ki67 was significantly decreased expressed compared with anti-PD1 + TKI group (Fig. 6D and E).These evidence indicated that AA enhanced the inhibition of HCC when combined with anti-PD1 + TKI therapy in vivo.

**Fig. 7:**
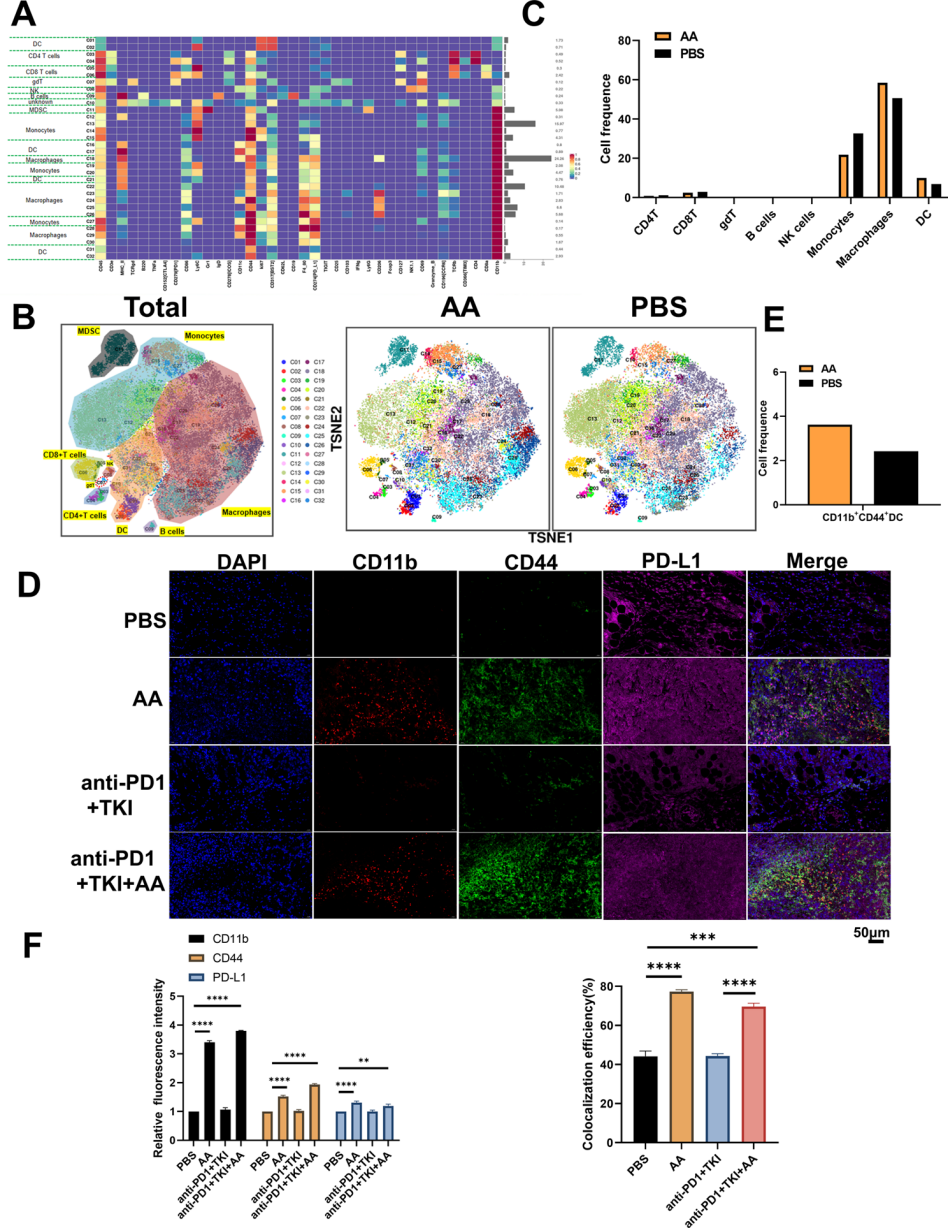
AA enhances the efficacy of anti-PD1 therapy in HCC by improving CD11b^+^CD44^+^PD-L1^+^ cell population. (**A**) The expression levels of various immune factors in PBS and AA group. The left side of the figure is labeled with the different cell populations, and the right side of the figure is labeled with the percentage of different cell populations. (**B**) The expression levels of various immune cells in PBS and AA group. (**C**) The proportion of immune cell populations in PBS and AA group. (**D**) Mouse tumor immunofluorescence(IF) CD11b, CD44, PD-L1 staining in PBS and AA group. Scale bar, 50 μm. (**E**) The population of CD11b^+^CD44^+^PD-L1^+^ cell in PBS and AA group. (**F**) Histogram of mouse tumor IF analysis in PBS and AA group. **, *P* < 0.01, ***, *P* < 0.001,****, *P* < 0.0001

**Fig. 8:**
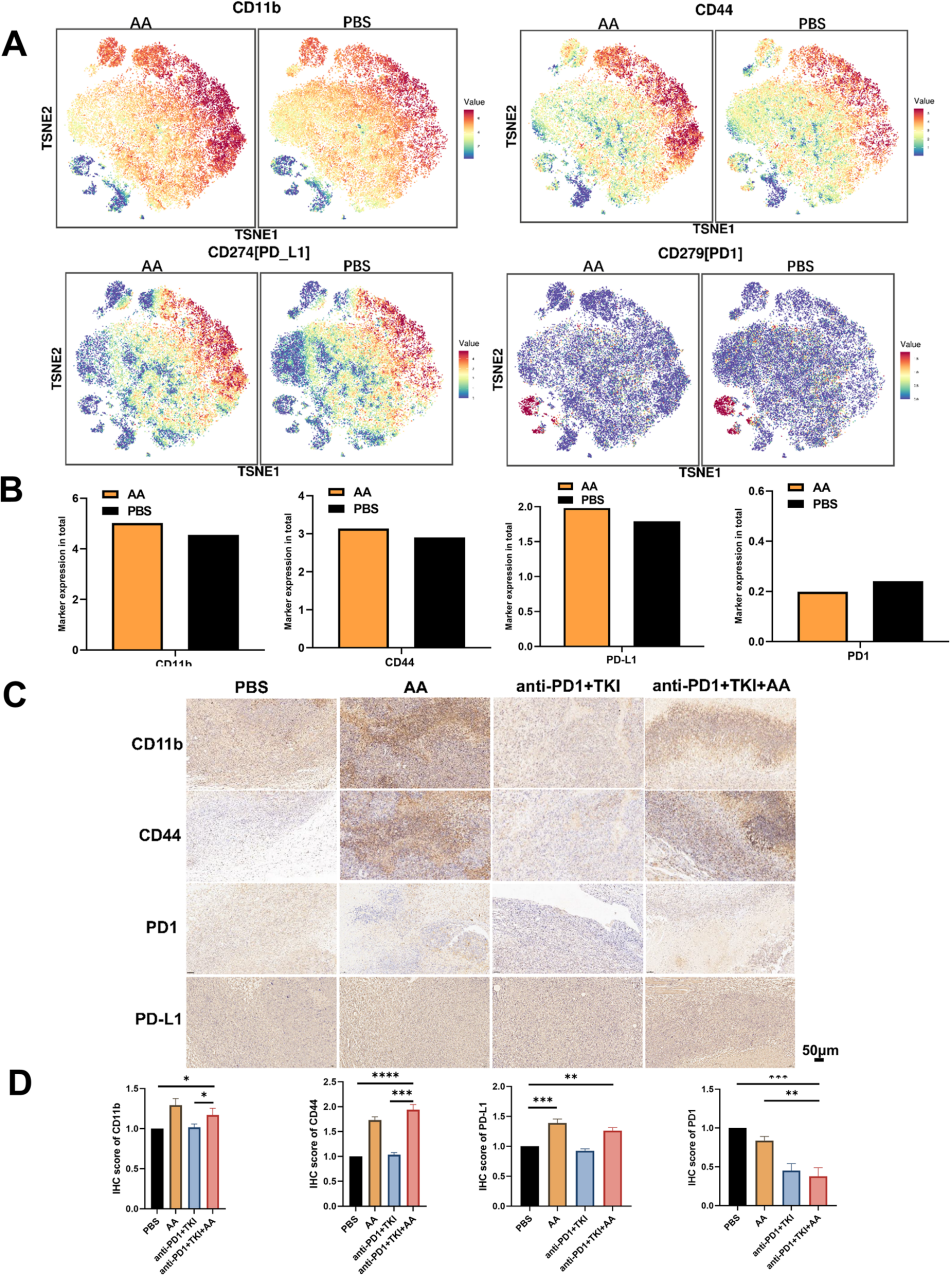
The expression of immune factor in PBS group and AA group. (**A**) The expression of CD11b, CD44, PD-L1,PD1 in PBS group and AA group. (**B**) Histogram of expression of various immune factors in PBS group and AA group. (**C**)Mouse tumor immunohistochemistry CD11b, CD44, PD1,PD-L1 staining in PBS group, AA group, anti-PD1 + TKI group, and anti-PD1 + TKI + AA group. Scale bar, 50 μm. (**D**)Histogram of mouse tumor immunohistochemical analysis in PBS and AA group

In order to explore the changes of the tumor immune microenvironment of HCC with addition of AA, two tumor samples from PBS and AA C57BL/6 mice were detected by mass cytometry, respectively.There were 32 cell clusters in total, and the respective cell clusters were defined based on the specific markers of the respective cell types (Figure S5, Fig. 7A and B). As indicated from the result, the relative proportion of DC and macrophages in AA group showed an increasing trend compared with the PBS group, whereas monocytes showed a downward trend (Fig. 7C). In particular, we found that the CD11b^+^CD44^+^PD-L1^+^ cell population was significantly increased in the AA group compared to PBS (Fig. 7D), which aroused our great interest. We further verified the mouse tumor tissue by immunofluorescence, and found that the expressions of CD11b^+^CD44^+^PD-L1^+^ were significantly increased in the AA group compared with the PBS group (Fig. 7E and F), which were consistent with the results of mass cytometry. In addition, it was revealed that the expression of CD11b, CD44, and PD-L1 increased while PD1 decreased in AA group (Fig. 8A and B), which was consistent with immunohistochemistry results(Fig. 8C and D). These results confirmed that AA could improve the anticancer effect of immunotherapy by recruiting CD11b^+^CD44^+^PD-L1^+^ cells.

## Discussion

Immunotherapy has opened a new chapter in cancer treatment, and monoclonal antibodies targeting PD1 have shown promising effectiveness as single agents or combination in the treatment of cancers including HCC [18]. Immune-related adverse events (irAEs) are largely affecting the skin, liver, gut, endocrine glands, lungs, and other organs as a result of the widespread usage of immune monoclonal antibodies. Liver injury is a serious adverse event. Up to 16% of individuals receiving immune checkpoint inhibitors(ICIs) experienced this occurrence. Although immunosuppressive agents such as steroids and immunomodulators can be used to treat irAEs of liver injury, their immunosuppressive effects will lessen the ability of immune monoclonal antibodies to stimulate the immune system to attack tumors [19, 20]. It is vital to identify a solution to the issue of the mechanism of liver injury brought on by irAEs and the mitigation of liver injury in HCC patients while assuring the activation of immune anti-tumor.

In liver injury, the increase of enzymes such as ALT and AST indicates that the death of liver cells caused by inflammation caused by immune cells involves the metabolic process of cells. Metabolomics technology has distinct advantages in research fields like disease mechanism and novel drug screening because it can more thoroughly detect and analyze metabolic changes in organisms, make it easier to find the relationship between metabolites, physiology and pathology, and more. In order to give guidance for research on liver protection, this study has employed metabolomics technology to examine liver tissues from HCC patients both before and after immuno-targeted treatment and screen for targeting differential metabolites. Using three analytical techniques, including overall analysis before and after treatment, pre- and post-treatment analysis for severe liver damage, and pre- and post-treatment analysis for mild liver damage, we performed differential metabolite analysis on samples before and after immuno-targeted treatment. We noticed that a few metabolites, such as isopalmitic acid, dodemorph, 3-hydroxybenzo(A)pyrene, N,N-dimethyldodecylamine N-oxide, AA, perfluoropropanesulfonic acid were either significantly different in the three assay. Bao et al. have shown that AA can alleviate liver injury caused by T cells [21]. AA, which is closely linked to antioxidant activity in liver injury, was considerably reduced in all three analysis according to our metabolomic findings. And this indicates that the amount of AA in the liver falls when the patients administer PD1 monoclonal antibody to harm the liver. According to previous research, AA may be able to reduce the effects of oxidative stress, which can harm the liver [22]. Moreover, some studies have shown that AA can act as an ferroptosis inhibitor [23]. And ferroptosis is important in the mechanism of liver injury [24]. Similarly, our results show that KEGG pathway analysis of samples before and after immuno-targeted treatment could be found to converge significantly in glutamate metabolism, biosynthesis of amino acids, and vitamin digestion and absorption, which were all simultaneously associated with ferroptosis [16, 17], and KEGG pathway analysis in the severe liver injury group after immuno-targeted treatment could be pooled in ferroptosis. Combined with a decrease of AA in all three analysis, it was of interest to explore the relationship between AA and ferroptosis in liver tissue.

In our investigation, immune therapy coupled with TKIs was used in vivo experiments to simulate as closely as possible the systemic therapy given to patients with advanced HCC. Our results showed that the anti-PD1 + TKI group could cause severe liver injury, however, the addition of AA to the anti-PD1 + TKI group alleviated PD1 + TKI-induced liver injury. The anti-PD1 + TKI + Fer-1 group and the PD1 + TKI + AA group had similar effects on alleviating liver injury, and western blotting experiments confirmed that AA could be the mitigation of liver injury by inhibiting hepatocyte ferroptosis. A new type of cell-controlled death known as ferroptosis is characterized by intensely iron-dependent lipid peroxidation. Iron metabolism, lipid metabolism, oxidative stress, and the creation of NADPH, GSH, and coenzyme Q10(CoQ10) are just a few of the biological processes that are involved in ferroptosis [25].Wang M et al. studied drug-induced liver injury has been associated with hepatocyte ferroptosis [26]. According to the research of Tsurusaki S et al., hepatocyte ferroptosis is crucial to the development of steatohepatitis [27]. These studies reveal an important relationship between liver injury and ferroptosis. Cysteine is used to produce glutathione, and it is obtained from the extracellular environment by the cystine transporter system Xc- (composed of the catalytic subunit SLC7A11 and the chaperone subunit SLC3A2) [28]. Cysteine is then converted to cysteine in the cytoplasm by a reduction reaction that consumes NADPH. In order to detoxify lipid peroxidation and to prevent ferroptosis, glutathione peroxidase 4 (GPX4) uses GSH [24]. The SLC7A11 and GPX4 protein expressions were all higher in the anti-PD1 + TKI + AA group compared to the anti-PD1 + TKI group, showing that hepatocyte ferroptosis was suppressed and that AA protected normal hepatocytes through the SLC7A11-GSH-GPX4 pathway. CCL_4_ created the LO2 hepatocyte injury model, and we used western blotting experiments, GSH and AA detection, and cellular ROS fluorescence experiments to demonstrate in vitro that AA protects hepatocytes.

Interestingly, in the present study, we also discovered that AA can boost the effect of anti-PD1 on tumor therapy when conducting in *vivo* experiments in which AA reduces liver injury. Luchtel RA et al. have indicated that AA increases the suppression of the anti-PD1 checkpoint, by increasing the intratumoral infiltration of macrophages and CD8^+^ T lymphocytes, the generation of granzyme B by cytotoxic cells (cytotoxic T cells and natural killer cells), and the release of interleukin 12 by antigen-presenting cells [12]. The combined AA group demonstrated a better tumor treatment effect than the other groups in the data of tumor growth volume and tumor weight in the mouse liver cancer subcutaneous tumor model. The mass cytometry results of AA group showed that there are more DC cells and macrophages than there are in the PBS group, particularly a considerable increase in the CD11b^+^CD44^+^PD-L1^+^ cell population. The findings of mouse tumor IHC and IF further supported the finding that the anti-PD1 + TKI + AA group had higher levels of CD11b and CD44 molecules. Both DC cells can express CD11b, CD44, and macrophages express CD11b [29], so we think that both are present in the CD11b^+^CD44^+^PD-L1^+^ cell population. DC cells are the most powerful antigen presenting cells in the immune system and are essential for adaptive immune responses. They usually trigger T cell responses within tumors, which slows the spread of disease and increases patient survival [30]. Duong et al. have revealed that CD11^+^ DC cells activate CD8^+^ T cells to promote anti-tumor immunity. This mechanism of activation of CD11b^+^ conventional DC cells characterized by the expression of interferon (IFN)-stimulated genes (ISG + DCs) and able to acquire and present intact MHC class I complexes of tumor-derived peptides was elucidated [31]. Yunge Gao et al. have showed that CD44^+^ DC cells can enhance immune antitumor effects in ovarian cancer by inhibiting the suppressive effect of growth differentiation factor-15 (GDF-15) on CD11c, CD83 and CD86 expression [32]. However, the CD11b^+^CD44^+^PD-L1^+^ cell population had high PD-L1 expression, which led to increased tumor immune escape. Studies have confirmed that DC cells are the main cells that produce PD-L1, and inhibition of PD-L1 expression in DC cells can reduce tumor growth [33]. In addition, macrophages in the CD11b^+^CD44^+^ PD-L1^+^ cell population can also produce PD-L1 [34]. Our study aims to prove that AA can recruit more CD11b^+^CD44^+^PD-L1^+^ cells, and even if its function is not good, it can still turn cold tumors into hot tumors, and then use PD1 monoclonal antibody to further enhance immune cells after increasing the expression of PD-L1.Elevated PD-L1 interacts with PD1 on CD8^+^ T cells, resulting in a reduced immune anti-tumor killing effect and PD-L1 expression can be an important marker for the effectiveness of immunotherapy [35, 36]. AA combined with PD1 monoclonal antibody increases CD11b^+^CD44^+^ PD-L1^+^ cell infiltration to enhance antigen presentation, resulting in an enhanced immune anti-cancer effect. Nevertheless, the sample size we tested is too small, and larger scale animal experiments as well as validation at the clinical level of the mitigating effect of AA on immuno-targeted therapy induced liver injury are needed.

## Conclusion

**Fig. 9:**
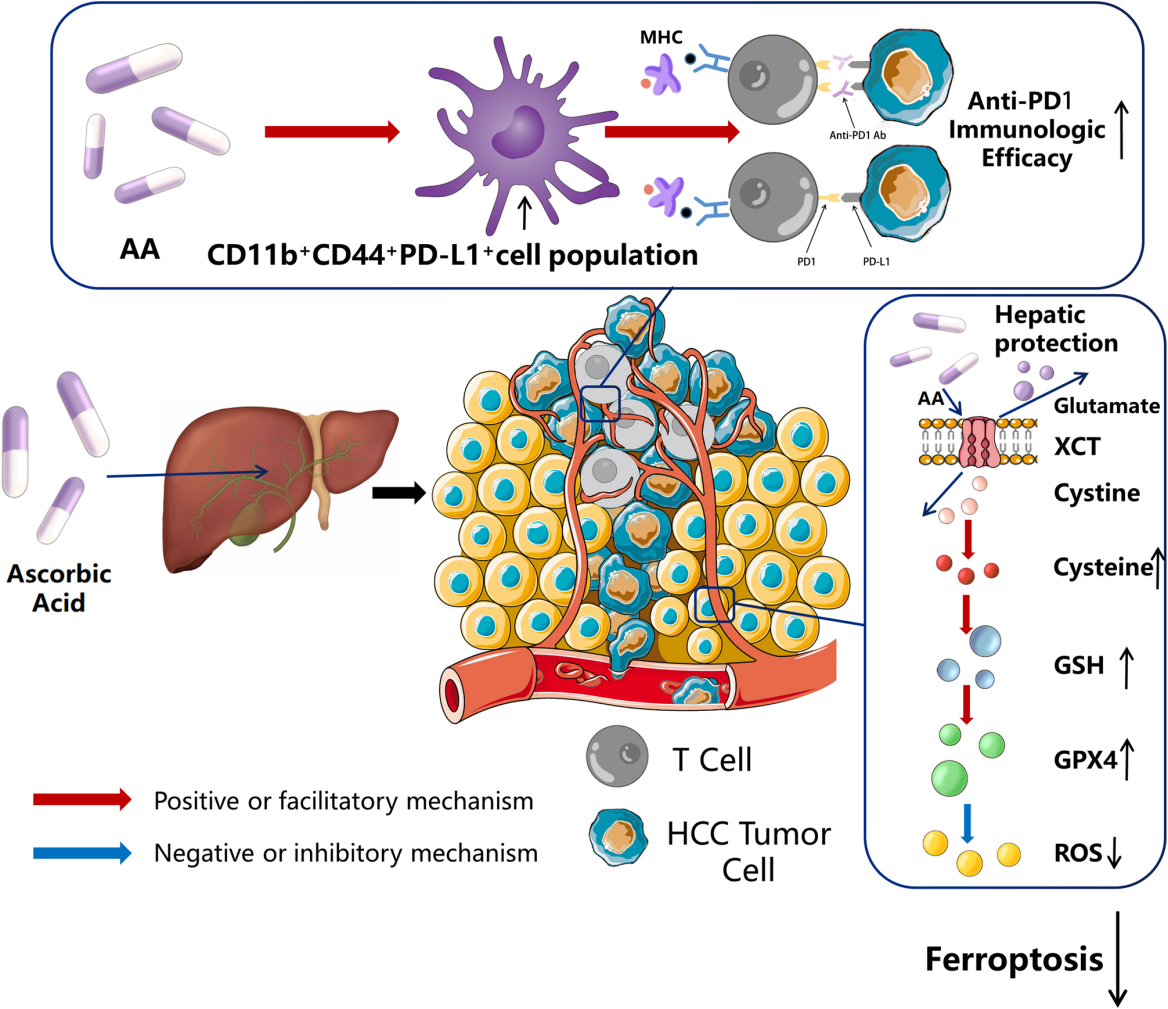
Schematic diagram of the mechanism relative to AA reduce liver injury by preventing hepatocyte ferroptosis and improve the immunotherapy effect of anti-PD1 by boosting CD11b^+^CD44^+^PD-L1^+^ cell population

Metabolomics results suggest that AA deficiency is present in liver injury caused by immunotherapy combined with targeted therapy in HCC. Subsequent in *vivo* and in *vitro* studies show that AA can reduce liver injury by preventing hepatocyte SLC7A11/GPX4 ferroptosis and improve the immunotherapy effect of anti-PD1 by boosting CD11b^+^CD44^+^PD-L1^+^ cell population (Fig. 9).

### Electronic supplementary material

Below is the link to the electronic supplementary material.

Supplementary Material 1

Supplementary Material 2

## Data Availability

The data that support the findings of this study are available from the corresponding author upon reasonable request.

## Abbreviations

HCC: Hepatocellular carcinoma
irAEs: Immune-related adverse events
AA: Ascorbic acid
TKI: Tyrosine kinase inhibitor
HE: Hematoxylin-eosin
PLS-DA: Partial least squares discrimination analysis
ICIs: Immune checkpoint inhibitors
AST: Aspartate aminotransferase
ALT: Alanine aminotransferase
GSH: Glutathione
GPX4: Glutathione peroxidase 4
SLC7A11: Solute carrier family 7 member 11
DC cell: Dendritic cell
IHC: Immunohistochemistry
IF: Immunofluorescence
