# Supplementary material for: Metabolomics reveals ascorbic acid inhibits ferroptosis in hepatocytes and boosts the effectiveness of anti-PD1 immunotherapy in hepatocellular carcinoma
Source: Cancer Cell Int. 2024 May 31;24:192. doi: 10.1186/s12935-024-03342-0 (PMC11143590; doi:10.1186/s12935-024-03342-0)

**Supplement figure legend：**

Figure S1: Metabolic difference map positive pattern analysis of liver tissue samples from severe liver injury before and after immune targeted therapy. (A) The circos diagram of differential metabolite. (B) Differential metabolite hierarchical clustering results, the redder the color, the higher the relative expression, and the bluer the bluer, the lower the relative expression. The metabolite classification information of the KEGG and HMDB databases is also shown in the figure. (C) Differential metabolites with a higher degree of importance are displayed, the abscissa is the log transformation of FC, and the ordinate is the metabolites. The blue and red dots on the left and right sides represent the down-regulated and up-regulated differential metabolites, respectively. (D) The pathways with the top30 significance are displayed, the abscissa represents the negative logarithmic transformation of p-value, the ordinate represents the name of the pathway, and the size of the circle represents count, that is, the number of differential metabolites annotated into the pathway; the color of the circle corresponds to the corrected p -value, more significant from red to blue. (E) Salient pathway histogram showing KEGG pathway enriched for salience top30. (F) The first column on the left of the significant pathway Sankey diagram represents the differential metabolites of up & down, and the width of the branch curve corresponds to the size of the data flow.


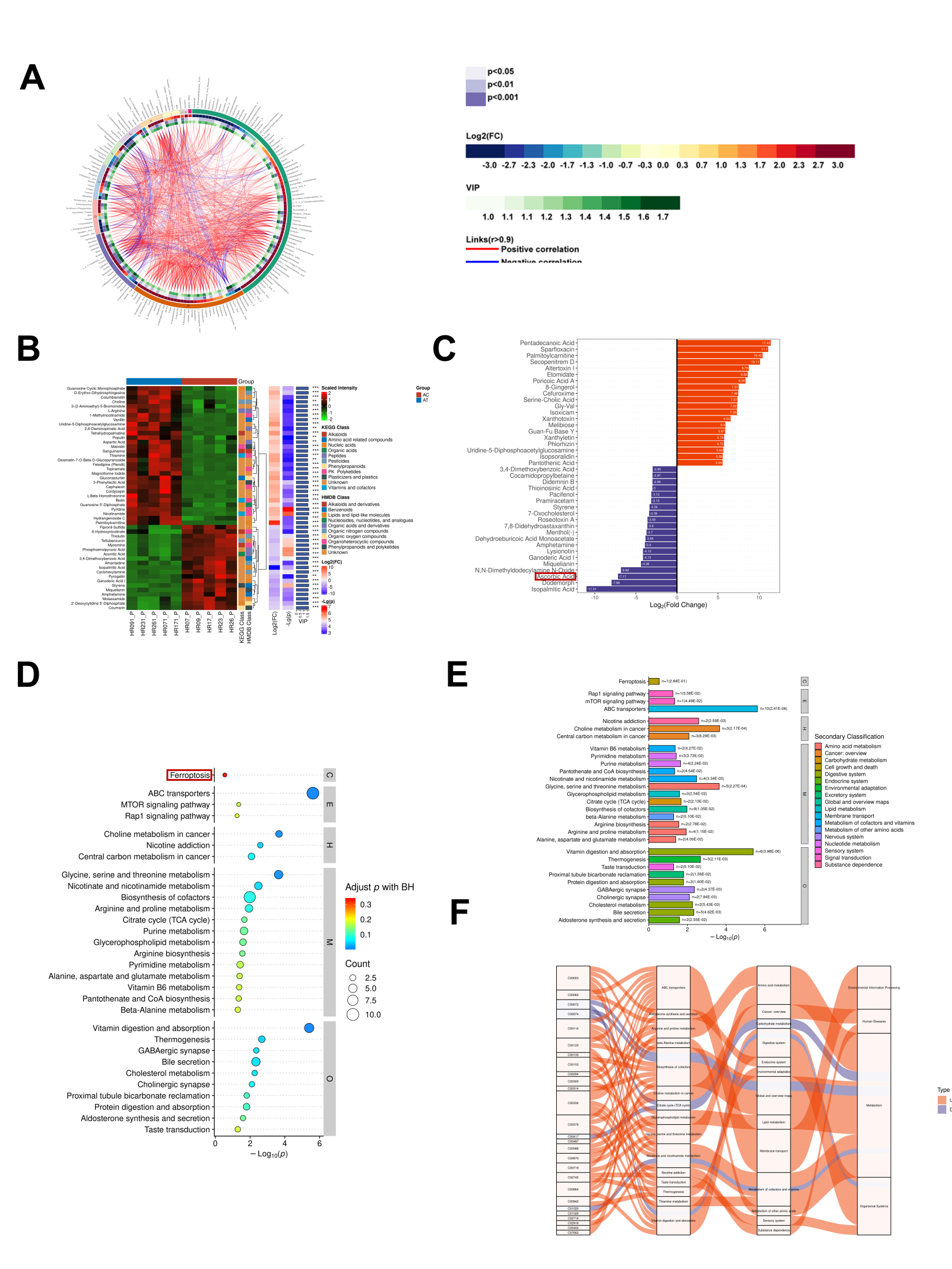


Figure S2: Metabolic difference map negative pattern analysis of liver tissue samples from severe liver injury before and after immune targeted therapy. (A) The circos diagram of differential metabolite. (B) Differential metabolite hierarchical clustering analysis. (C) Differential Metabolite Importance Analysis.(D) The prominent top30 pathway salient pathway bubble chart. (E) Salient pathway histogram showing KEGG pathway enriched for salience top30. (F) Significant pathway Sankey diagram.


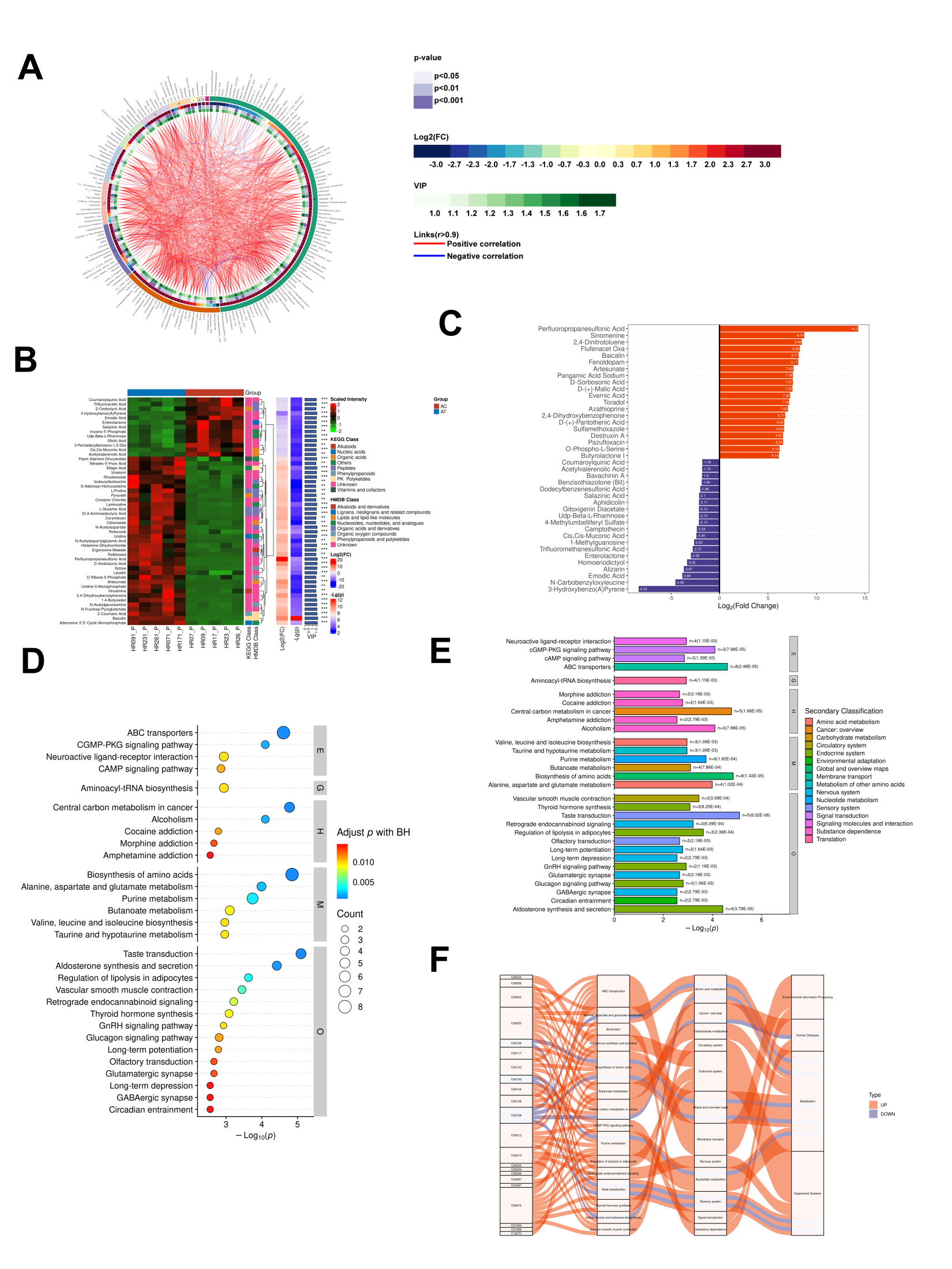


Figure S3: Metabolic difference map positive pattern analysis of liver tissue samples from mild liver injury before and after immune targeted therapy. (A) The circos diagram of differential metabolite. (B) Differential metabolite hierarchical clustering analysis. (C) Differential Metabolite Importance Analysis. (D) The prominent top30 pathway salient pathway bubble chart. (E) Salient pathway histogram showing KEGG pathway enriched for salience top30. (F) Significant pathway Sankey diagram.


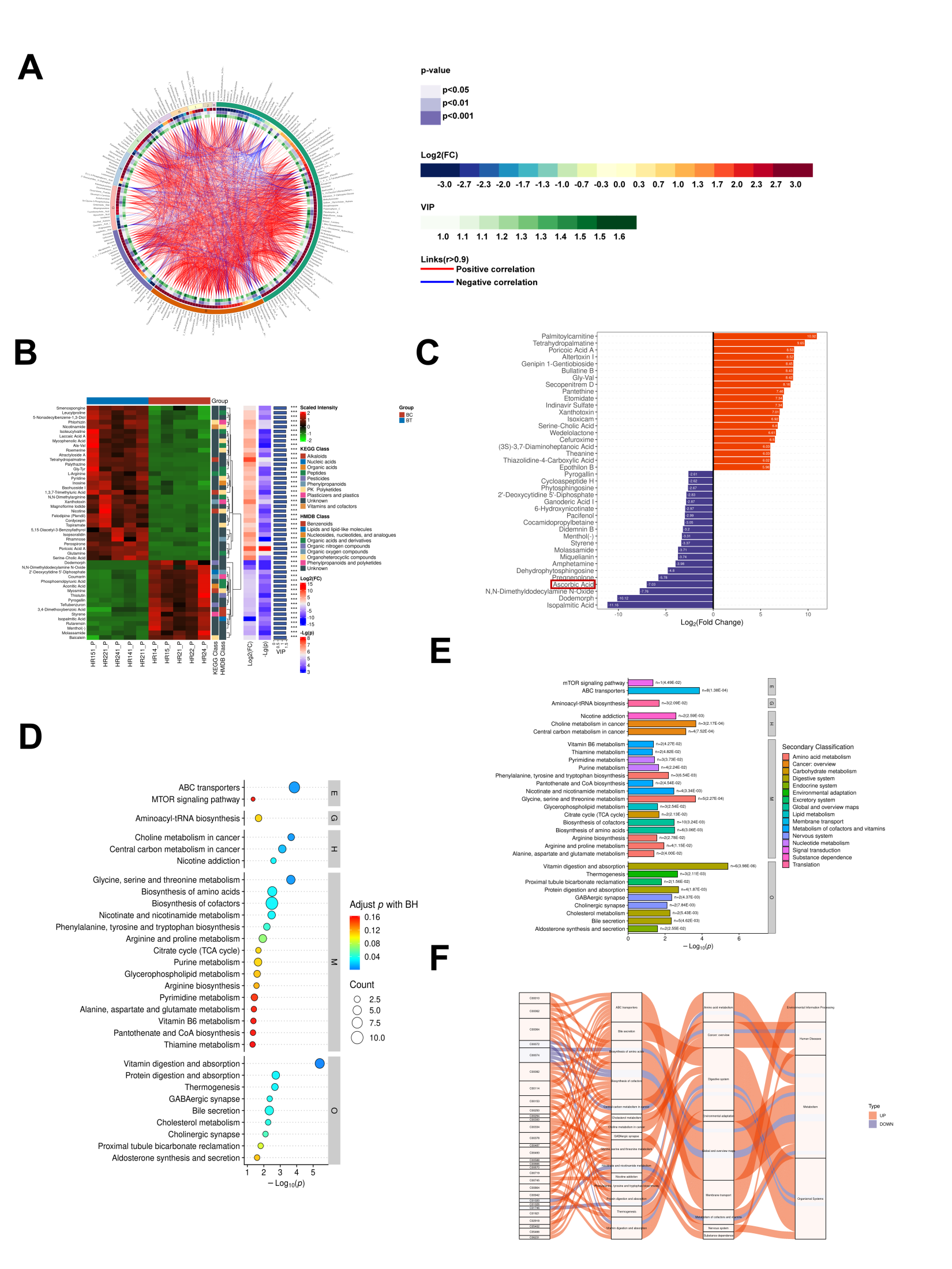


Figure S4: Metabolic difference map negative pattern analysis of liver tissue samples from mild liver injury before and after immune targeted therapy. (A) The circos diagram of differential metabolite. (B) Differential metabolite hierarchical clustering analysis. (C) Differential Metabolite Importance Analysis. (D) The prominent top30 pathway salient pathway bubble chart. (E) Salient pathway histogram showing KEGG pathway enriched for salience top30. (F) Significant pathway Sankey diagram.


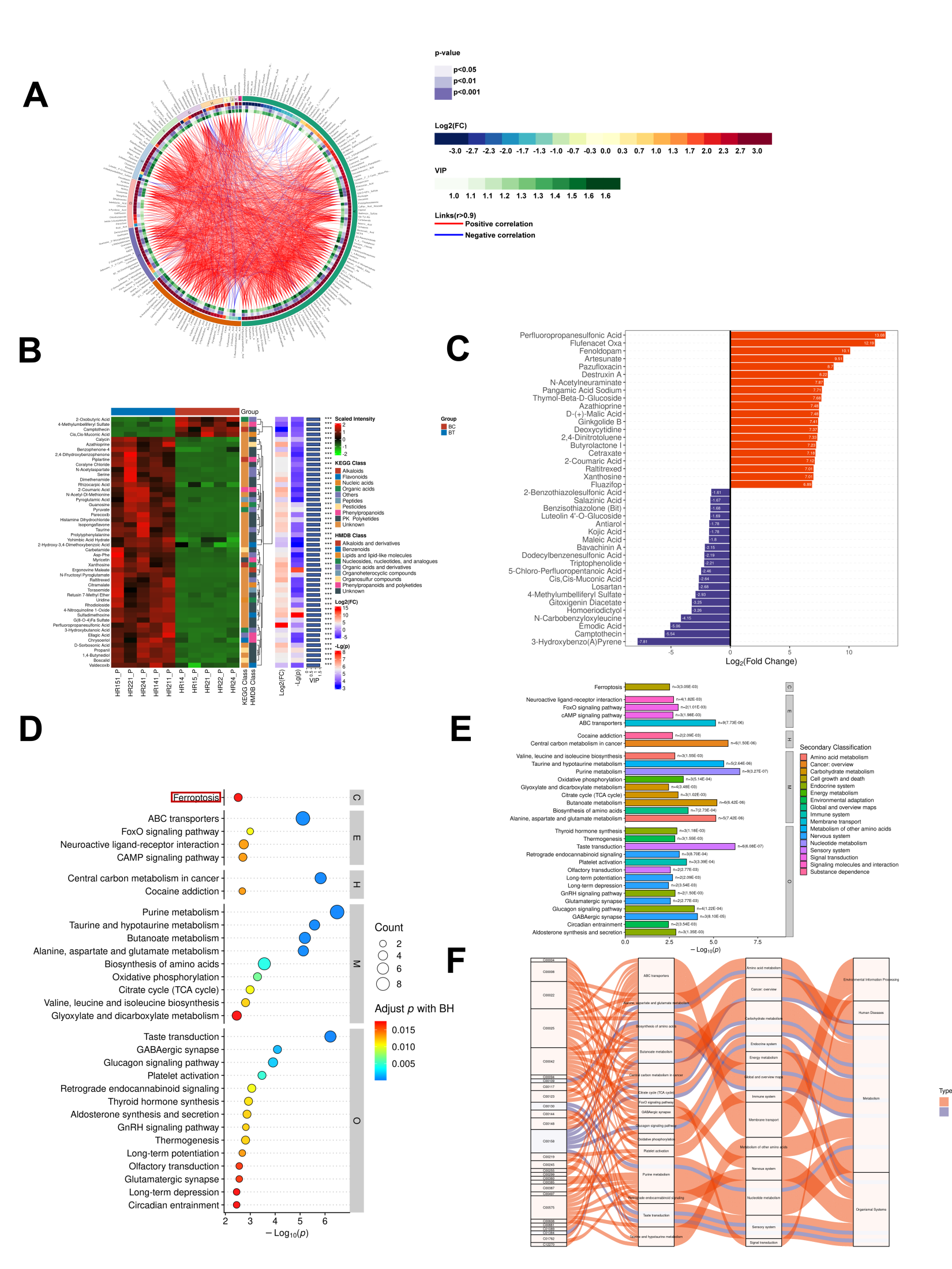


Figure S5: The expression of immune depletion factor in PBS group and AA group.


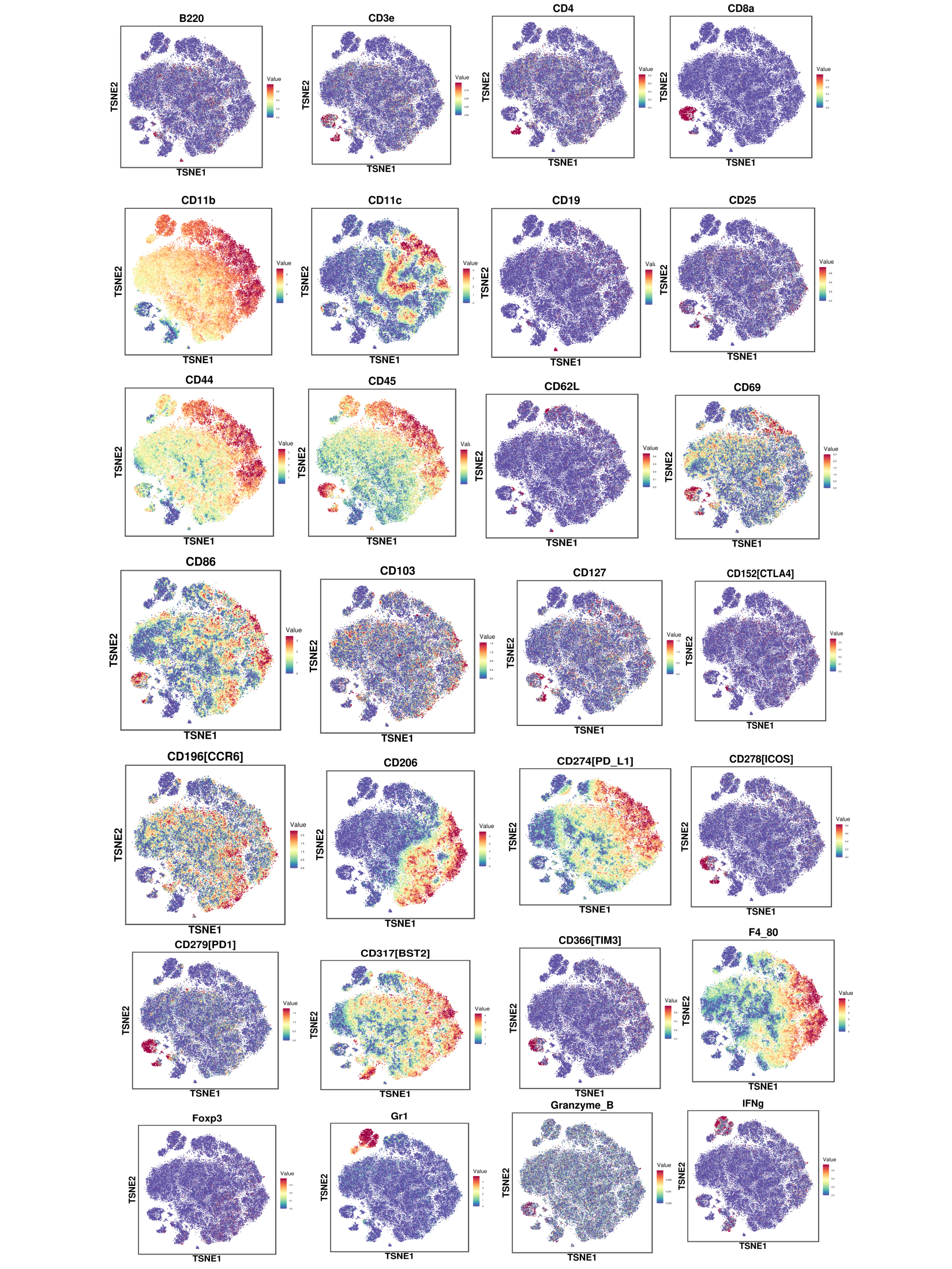

Supplement: Supplementary file 1 — Supplementary Material 1 [file 12935_2024_3342_MOESM1_ESM.docx]
