# Supplementary figures and images for: Metabolomics reveals ascorbic acid inhibits ferroptosis in hepatocytes and boosts the effectiveness of anti-PD1 immunotherapy in hepatocellular carcinoma
Source: Cancer Cell Int. 2024 May 31;24:192. doi: 10.1186/s12935-024-03342-0 (PMC11143590; doi:10.1186/s12935-024-03342-0)

Figure 4

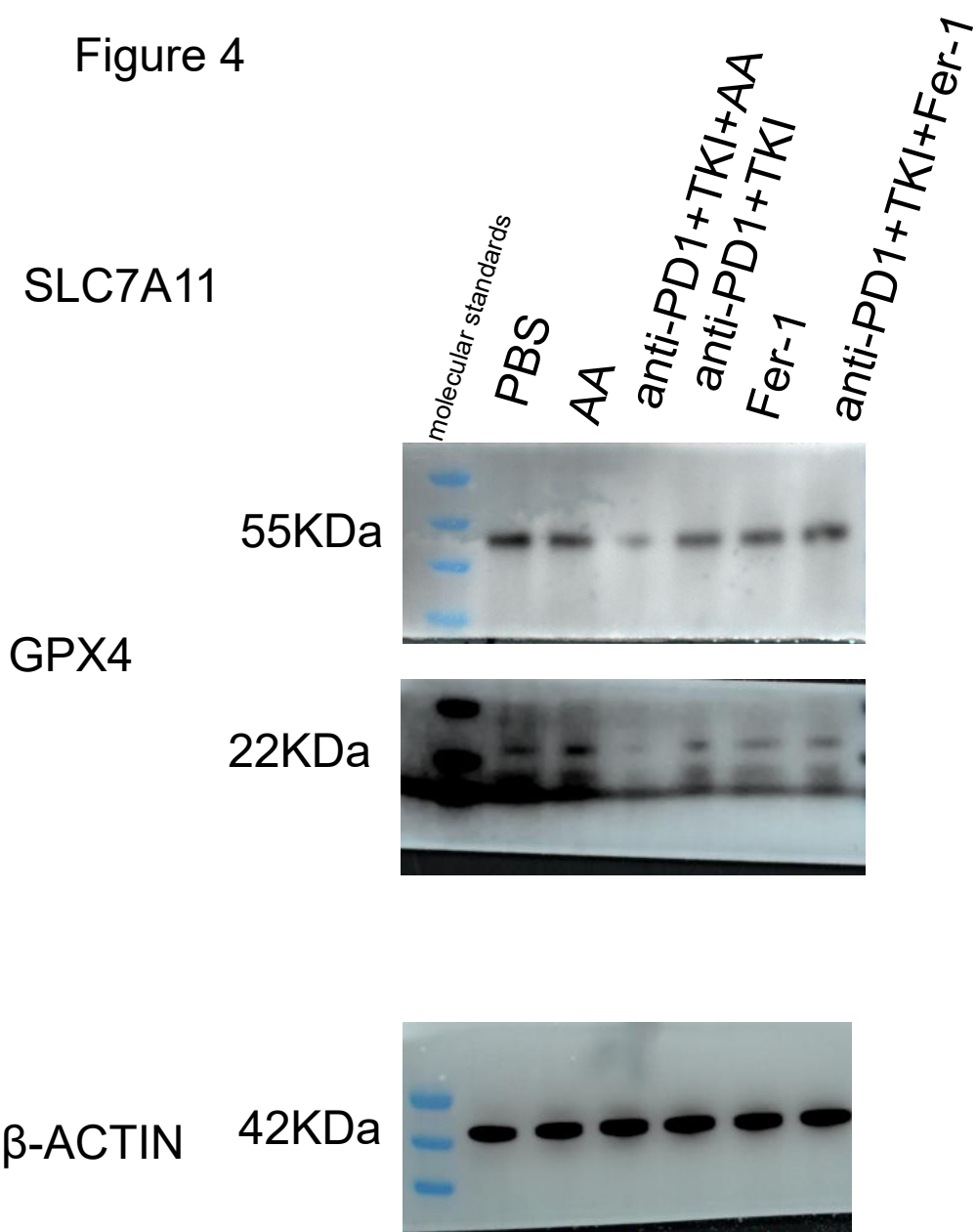

Figure 5

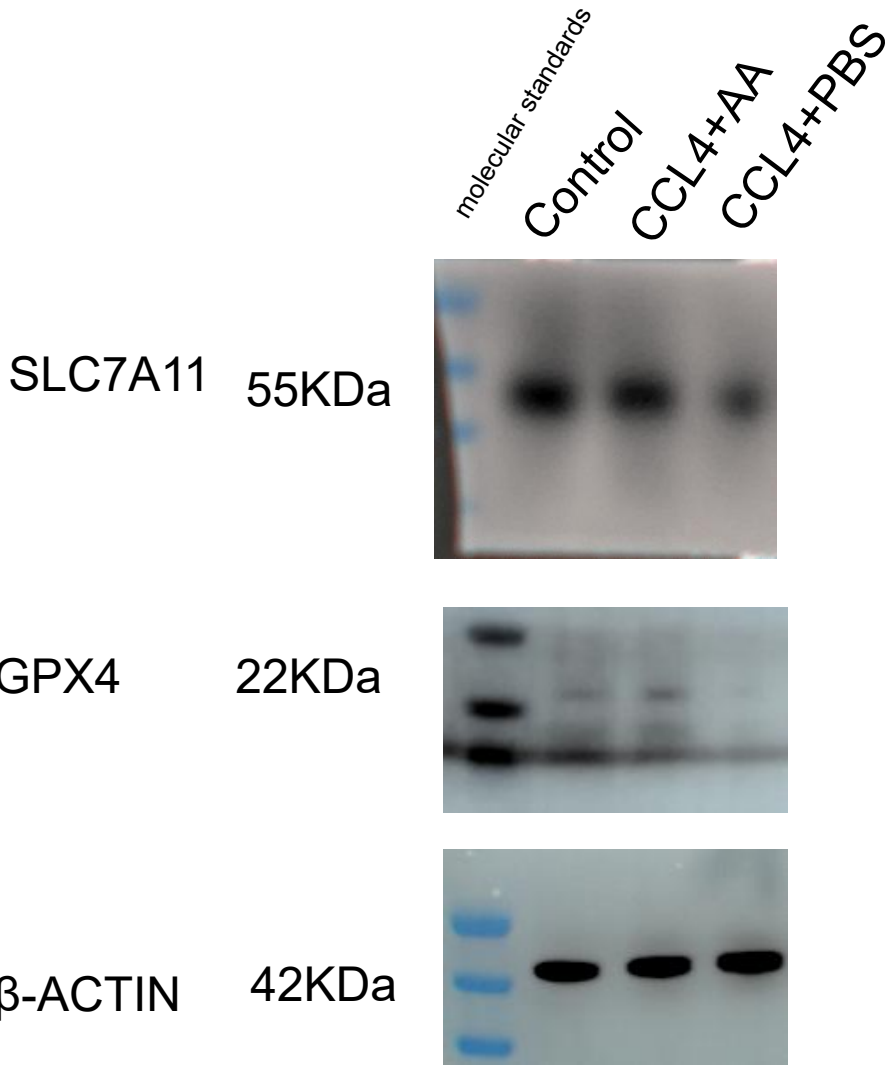

Supplement: Supplementary file 2 — Supplementary Material 2 [file 12935_2024_3342_MOESM2_ESM.pdf]
